# Individual Human Brain Areas Can Be Identified from Their Characteristic Spectral Activation Fingerprints

**DOI:** 10.1371/journal.pbio.1002498

**Published:** 2016-06-29

**Authors:** Anne Keitel, Joachim Gross

**Affiliations:** Centre for Cognitive Neuroimaging, Institute of Neuroscience and Psychology, University of Glasgow, Glasgow, United Kingdom; University Medical Center Hamburg-Eppendorf, GERMANY

## Abstract

The human brain can be parcellated into diverse anatomical areas. We investigated whether rhythmic brain activity in these areas is characteristic and can be used for automatic classification. To this end, resting-state MEG data of 22 healthy adults was analysed. Power spectra of 1-s long data segments for atlas-defined brain areas were clustered into spectral profiles (“fingerprints”), using *k*-means and Gaussian mixture (GM) modelling. We demonstrate that individual areas can be identified from these spectral profiles with high accuracy. Our results suggest that each brain area engages in different spectral modes that are characteristic for individual areas. Clustering of brain areas according to similarity of spectral profiles reveals well-known brain networks. Furthermore, we demonstrate task-specific modulations of auditory spectral profiles during auditory processing. These findings have important implications for the classification of regional spectral activity and allow for novel approaches in neuroimaging and neurostimulation in health and disease.

## Abstract 2

Oscillatory activity in anatomically defined brain areas is organized according to several different spectral modes; these modes are characteristic and can be used for automatic classification.

## Introduction

In the human brain, anatomy constrains and shapes function, both at the local and global level [1]. Individual brain areas can be differentiated either locally, based on their characteristic cytoarchitectonic structure [2,3], or globally, based on their characteristic anatomical connectivity. These properties are thought to underlie both the functional specialisation of individual areas as well as their integration into large-scale functional brain networks. Many studies in the past decade have identified large-scale resting-state brain networks in fMRI recordings [4–6]. These networks are characterised by the synchronized activity of their constituent nodes. In electroencephalographic and magnetoencephalographic (EEG/MEG) recordings, similar networks emerge from brain areas that act in concert, as expressed in correlations of spectral power over time, predominantly at frequencies between 10 Hz and 30 Hz [7–13]. These networks are likely involved in the regulation of physiological and pathological activity (for reviews, see [14–16]). Interestingly, recent research suggests that the coordinated network activity in the brain during tasks is to some extent already reflected in intrinsic, ongoing activity during rest [1,17–19]. Because the dynamics of resting-state activity reflect the functional architecture of cortical networks [20], it is an ideal starting point for the analysis of general brain function.

While studying the global network level has dramatically improved our understanding of functional resting states, we still know surprisingly little at the level of individual brain areas. The brain’s cytoarchitectonic structure and specific anatomical connectivity suggests local specificity. But how is this reflected in the ongoing activity of individual brain areas? Does the characteristic local and global anatomical structure lead to distinct neural activity patterns in individual areas during rest?

In previous electrophysiological research, neural resting-state “fingerprints” that have often been reproduced comprise the parieto-occipital alpha rhythm (7–13 Hz) and the sensorimotor mu rhythms (7–13Hz, 18–30Hz) [10,21]. Brain oscillations at other frequencies have been found to be more dispersed and inconsistent in the brain, although theta (3–7Hz) and delta (1–3 Hz) are often prominent in anterior areas [10,22,23]. These results suggest characteristic frequencies in a subset of broader areas while also suggesting large variability.

The first aim of the present research was to comprehensively characterise intrinsic, ongoing spectral activity of anatomical areas during rest. Two complementing clustering approaches (*k*-means and Gaussian mixture [GM] modelling) were used within and across individuals to identify characteristic oscillatory patterns (“spectral profiles”) within anatomically defined individual brain areas (AAL, Automated Anatomical Labeling [24,25]). Previous studies have often presented average power spectra, which does not take into account frequency changes over time. We considered the nonstationarity of the brain by analysing single-trial as opposed to average frequency spectra.

The second aim was to test the regional specificity of these spectral profiles. Specifically, we investigated how reliably a brain area can be automatically classified from its resting-state oscillatory activity. This analysis was based on the computation of area-specific models of resting-state profiles from “training data” (i.e., data of half of our sample). Subsequently, “test data” (i.e., data from the remaining individuals) were used to automatically assign oscillatory activity to brain areas and statistically evaluate the performance of this classification. The spectral profile of one area is considered characteristic if test data were best assigned to the correct training model area.

## Results

### Computing Area-Specific Spectral Profiles

Standard spectral analysis of continuous electrophysiological data (resting state or continuous task) typically relies on variants of Welch’s method [26] that leads to a single power spectrum representing neural activity across the recording session. Such an approach suffers from four major shortcomings. First, EEG/MEG sensor recordings contain a linear superposition of contributions from different brain areas. This hinders accurate identification of spectral components specific to individual brain areas. Second, by representing neural activity in a single power spectrum, it ignores the dynamic nature of brain activity (e.g., [8,20,27]). Third, the power spectrum of electrophysiological activity is characterised by a power-law behaviour with a dominance of low frequencies (1/f characteristic) and strong ongoing 10-Hz activity (alpha rhythm) that complicates identification of frequencies other than alpha. Fourth, representing power spectra at the group level across participants is notoriously difficult due to the large interindividual variability and the absence of validated normalisation procedures.

**Fig 1:**
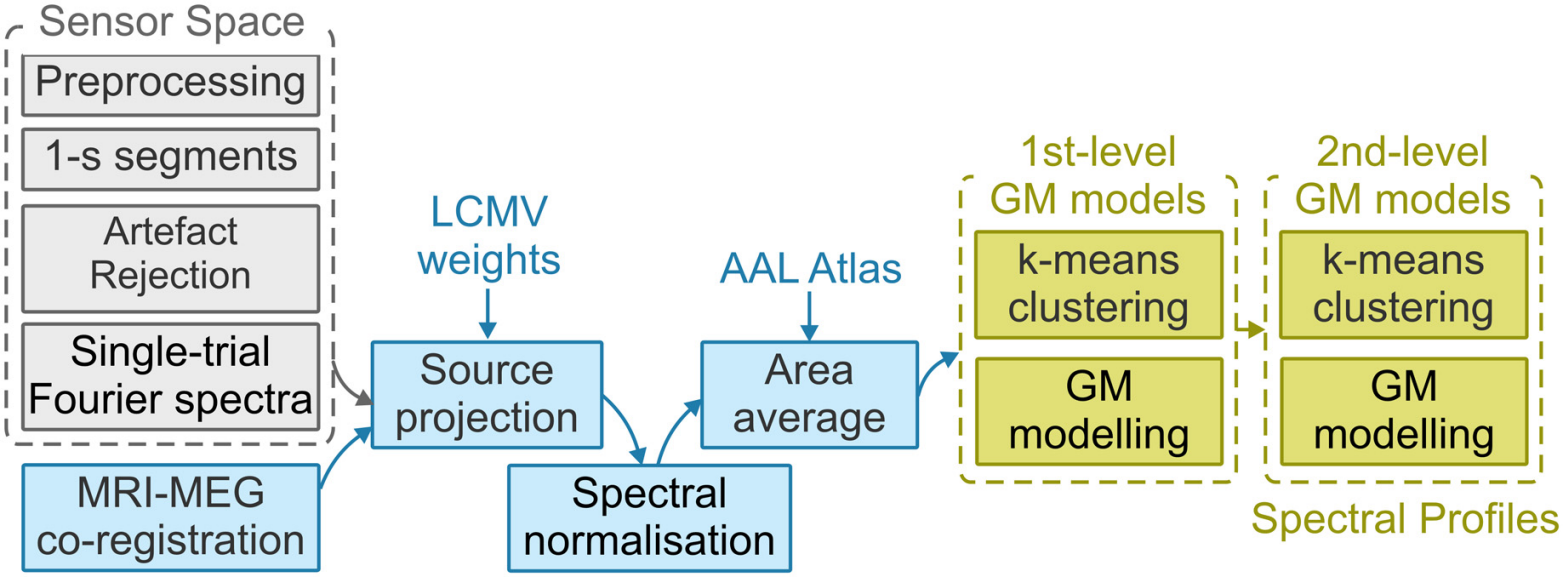
Analysis pipeline. After general preprocessing of the MEG data, the ~7-min continuous data were first segmented into 1-s segments. Artefactual segments and channels were rejected, and complex Fourier spectra were computed for each segment. Single trials (refers to 1-s segments) were then projected into source space by using previously computed linear constraint minimum variance (LCMV) weights. Data were spectrally normalised by dividing the spectrum of each segment and voxel by the average power spectrum across all segments and voxels per participant (ratio normalisation). Voxels were grouped according to the Automated Anatomical Labeling (AAL) atlas and activity averaged for each area. *k*-means and Gaussian mixture (GM) modelling algorithms were applied to reduce the dimensionality of trials for each participant (1st-level models, subject level) into ten distinct clusters. These ten clusters were clustered across all 22 participants, again using *k*-means and GM modelling algorithms (2nd-level models, group level). For 2nd-level models, the optimal number of clusters per area was computed, using a Silhouette criterion. Second-level GM models are referred to as spectral profiles or fingerprints.

We have developed an analysis pipeline to address these shortcomings by means of a novel combination of existing and validated methodologies (Fig 1). First, we performed spectral analysis at the level of standard anatomical parcellations of the human brain [24]. For this step, we projected MEG resting-state data into source space using established beamforming techniques [28–30]. Second, to account for the dynamic nature of brain activity, we analysed power spectra that corresponded to short segments of data (1 s) separately for each brain area. Third, to emphasise regionally specific spectral profiles, we expressed each single-segment power spectrum for each brain area as ratio to the mean power spectrum (averaged across all segments and brain areas). Fourth, we reduced the dimensionality of our data by computing GM models for each individual participant and brain area. Finally, individual GM models were clustered at the group level for each brain area using a second-level GM model. Only clusters found in the majority of participants (i.e., *N* = 16) were considered as stable.

### Spectral Profiles Consist of Multiple Spectral Modes

**Fig 2:**
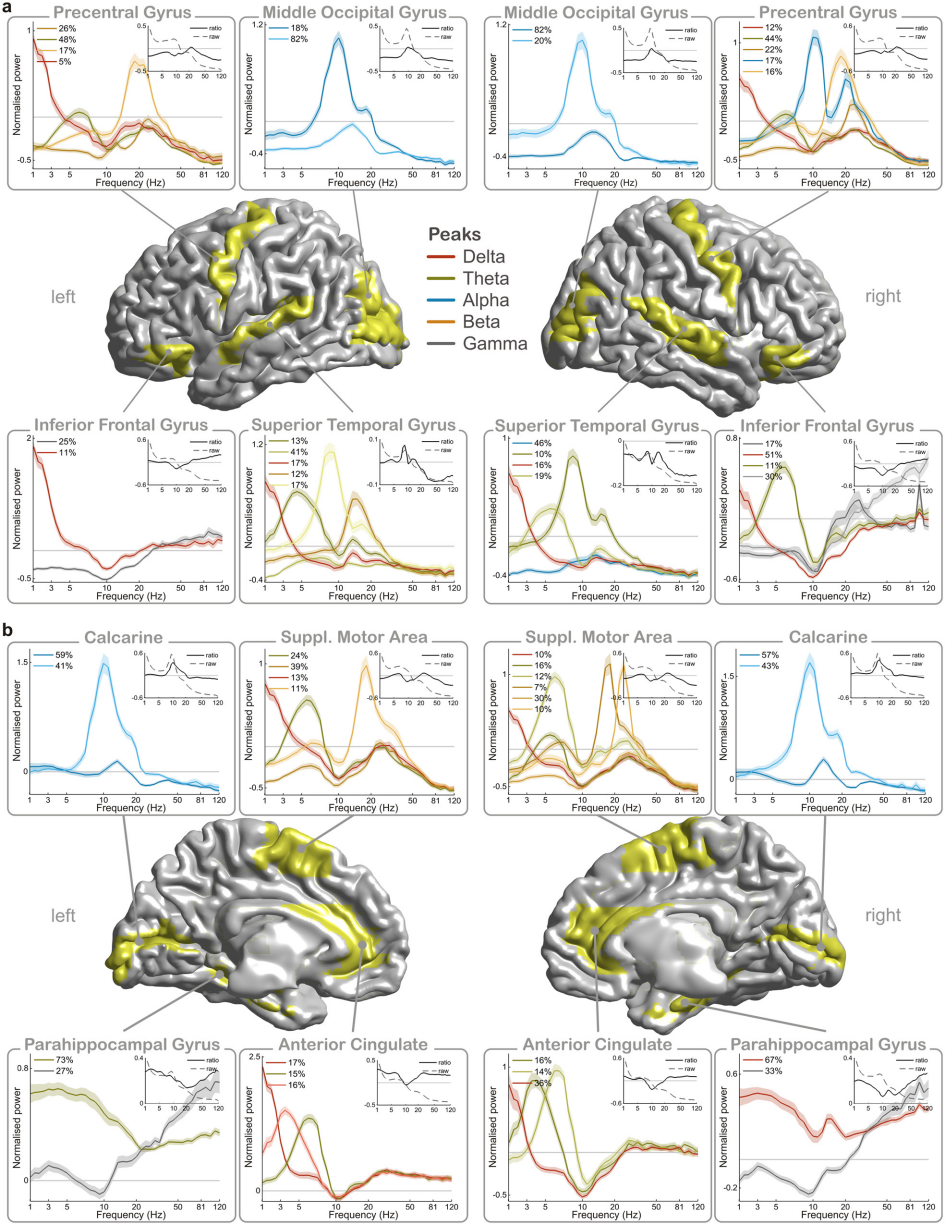
Spectral profiles of 16 example areas seen a) laterally and b) from the midsagittal plane. Clustered power spectra in source space represent normalised power, i.e., spectral power in comparison to the whole brain. Legends show the corresponding duration of each pattern (i.e., the percentage of trials in which each spectrum was present on average during recording). Shaded error bars illustrate the standard error of the mean across participants. The lines are colour-coded for the respective frequency bands (red: delta, green: theta, blue: alpha, orange: beta, grey: gamma). Inlets show average power spectra for respective areas without normalisation (dotted lines) and with ratio normalisation (continuous lines). Note that frequency on the *x*-axis is scaled logarithmically, and that data at 50 Hz (line noise) is interpolated in the plots.

Our analysis pipeline results in a number of spectra that best describe the brain activity in a given brain area across all 22 participants. We refer to these spectra as the “spectral profile” of a brain area. Fig 2 shows spectral profiles for 16 exemplary areas on the cortical surface. (results of all 115 atlas-defined areas can be found in S1–S10 Figs). The spectral profiles of individual brain areas consist, in general, of more than one spectrum (spectral activity in 99.1% of areas is best expressed by more than one cluster). Each spectrum has at least one characteristic peak. Thus, each brain area engages in several spectral “modes.”

### Spectral Profiles Are Characteristic for Individual Brain Areas

**Fig 3:**
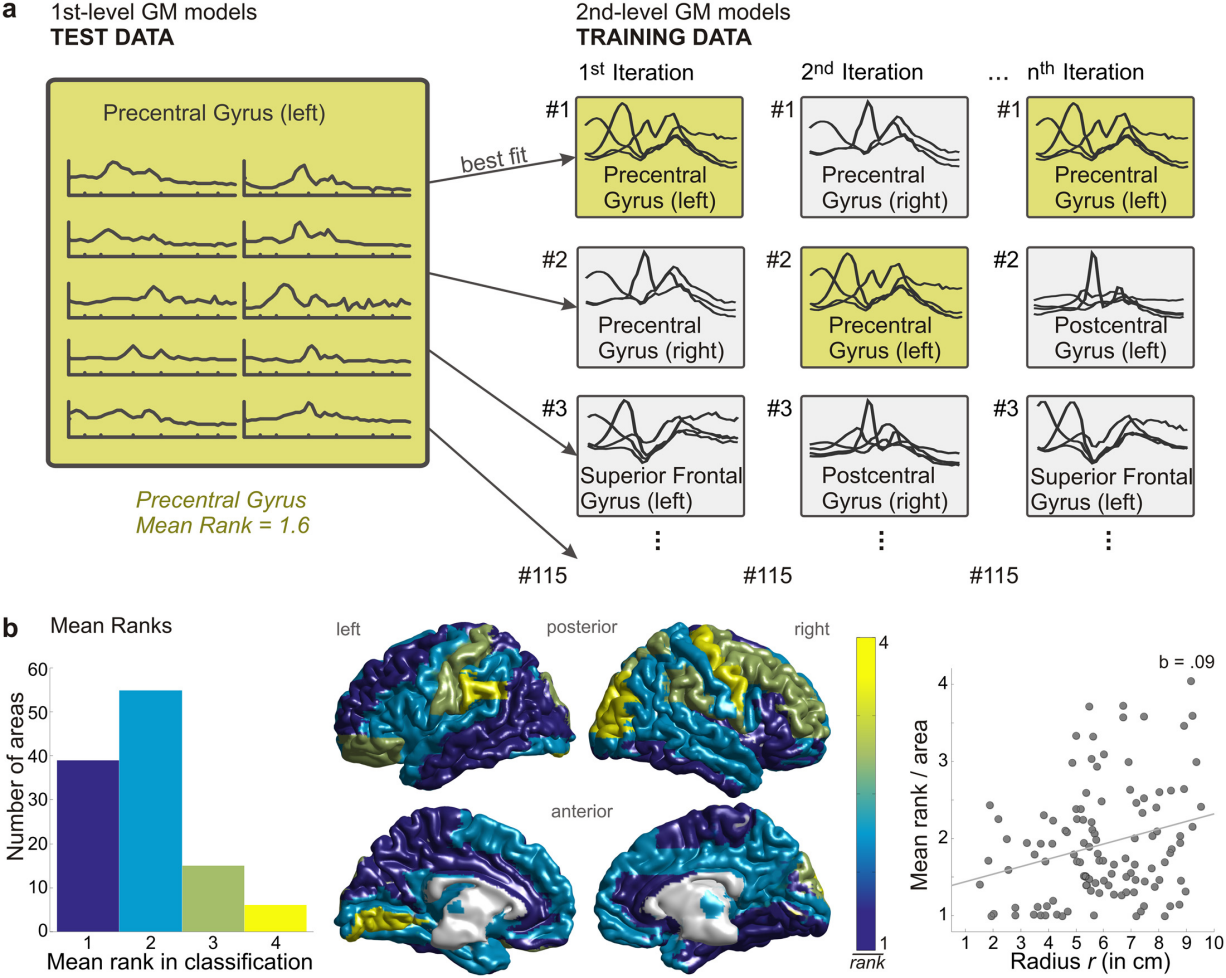
Classification procedure and results. a) Illustration of classification procedure. Second-level GM training models were created for all brain areas with data from half of the sample (group-level, right panel). Area-specific first-level GM test data from the remaining sample (subject-level, left panel) were then tested against each area-specific GM training model. The fit of each test set to each GM training model (determined through negative log-likelihood) was ranked. This was repeated 120 times, and the position of the correct area was averaged across iterations. For the illustrated example, the mean rank equalled 1.6 across 120 iterations. b) Distribution of mean ranks in classification. (left) Histogram of mean ranks across all 115 brain areas. Bin width is one. (middle) Topography of mean ranks (colour-coded from 1, blue, to 4, yellow; bin width is one). (right) Linear regression revealed a dependency of mean rank per area and the radius *r* from the centre of the brain. The mean rank increases the further an area is away from the centre of the brain. In other words, areas closer to the centre of the brain are easier to classify than more superficial areas. Data underlying this plot can be found in S1 Data.

To test the specificity of spectral profiles, we randomly split participants in two groups and computed area-specific spectral profiles (GM training models) for the first group. Data from each brain area from the second (test) group were then tested against each area-specific GM model from the training set. The fit of each test set to each GM training model was expressed in terms of its probability (determined through negative log-likelihood). These probabilities were then ranked, where a rank of 1 indicates that the correct model area was the most likely to fit the test area; a rank of 2 indicates it was the second most likely, and so on (see Fig 3a for illustration). The smaller the rank, the easier to classify an area is. We repeated this testing procedure 120 times with randomly drawn samples and computed the mean rank across iterations. A mean rank of 1 indicates that the correct area was assigned in 100% of iterations. The correct area was typically assigned in first or second place, resulting in a mean rank of 1.8. Also considering homologue areas in the other hemisphere as correct assignments improved the mean rank to 1.4. This indicates that individual brain areas can be identified with high accuracy based on their resting-state oscillatory activity.

A histogram and spatial distribution of the mean rank per area are illustrated in Fig 3b. The mean rank appears to be smaller near the centre of the brain as opposed to the cortical surface. Systematic linear regression analyses for spherical coordinates of brain regions and the mean rank per area revealed that the pattern is significantly related to the radius *r* from the centre of the brain. The mean rank decreases the closer an area is to the centre of the brain, *R*^2^ = .07, *F*(1,113) = 8.15, *p*_corrected_ = .04 (all *p*-values in this and the following sections are corrected for multiple testing using the sequential Bonferroni procedure) [31]. In other words, areas near the centre of the brain are slightly easier to classify than areas near the cortical surface. Overall, the high accuracy of the classification procedure illustrates that spectral estimates of anatomically defined brain areas have a characteristic fingerprint that can be used for automatic classification. Importantly, training and classification were performed on different data sets. Our results therefore demonstrate that the characteristic spectral profiles represented by GM models generalise across participants.

### Networks Consist of Brain Areas with Similar Spectral Profiles

**Fig 4:**
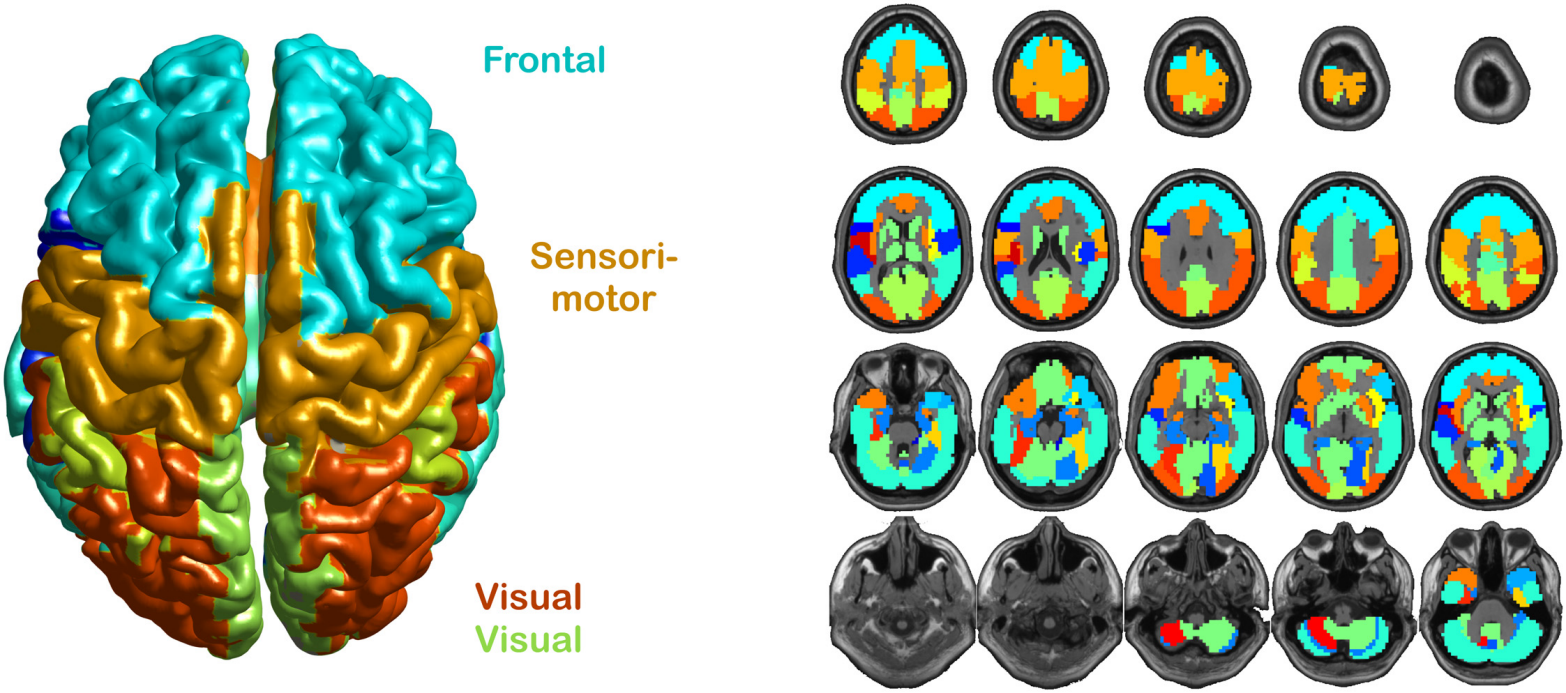
Area networks according to similarity analysis. (left) Topography of similar areas. (right) Slice view of the brain, including the cerebellum, with colour-coded similarity. A hierarchical clustering analysis (using negative log-likelihood between areas as distance measure) revealed several groups of areas that resemble previously identified brain networks. The frontal network (light blue) consists of bilateral medial and dorsolateral superior frontal gyrus, middle frontal gyrus, triangular inferior frontal gyrus, and right opercular inferior frontal gyrus. The sensorimotor network (orange) consists of bilateral pre- and postcentral gyri, paracentral lobule, and supplementary motor area. Two visual networks were found, a medial visual network (green) consisting of bilateral calcarine, cuneus, and precuneus and a parieto-occipital visual network (red) consisting of bilateral superior, middle, and inferior occipital gyri, superior parietal gyri, and supramarginal and angular gyri.

After having demonstrated that spectral profiles are characteristic, we investigated whether similarities exist that correspond to anatomical subdivisions of human brain areas. To this end, we used clustering based on the negative log-likelihood of the 2nd-level GM model as a measure of distance between areas. The negative log-likelihood quantifies in a graded manner the likelihood that data from one area are consistent with a GM model from another area. A hierarchical clustering approach was then used to find subsets of similar areas (see Fig 4; the full cluster tree, or dendrogram, can be found in S11 Fig). Although clustering is purely based on functional data (without using anatomical location), the algorithm linked subsets of bilateral frontal, central, and (two sets of) occipital areas. These functional clusters correspond well to large-scale anatomical parcellations of the human cortex.

### Number of Clusters per Anatomical Area

**Fig 5:**
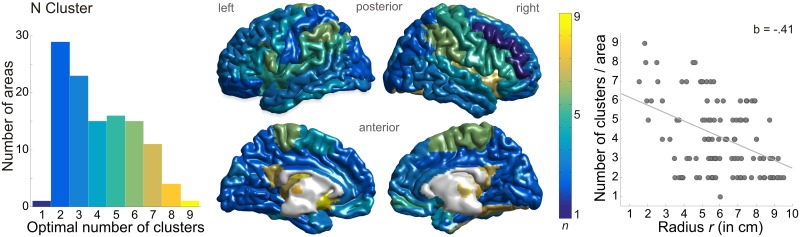
Distribution of number of clusters. (left) Histogram of cluster numbers across all 115 brain areas. Bin width is one. (middle) Topography of cluster numbers (colour-coded from 1, blue, to 9, yellow; bin width is one). (right) Linear regression reveals a dependency of number of clusters per area and the radius *r* from the centre of the brain. The number of clusters decreases with increasing radius. Data underlying this plot can be found in S2 Data.

We further aimed to characterise spectral profiles regarding number of clusters and peak frequencies (see supporting information S1 Text and S12 Fig for analysis of peak frequencies). The optimal number of clusters for each anatomical area was determined by a Silhouette criterion (see Materials and Methods). The average number of clusters was 4.10 ± 1.86 (*M* ± *SD*) and ranged between 1 and 9 per area (see Fig 5). A distribution of cluster numbers in anatomical areas illustrates that the cortical surface tends to have a low number of clusters, whereas the number seems to be more variable in subcortical regions. Systematic linear regression analyses for spherical coordinates of brain regions and the number of clusters per area reveals that the pattern is significantly related to the radius *r* from the centre of the brain. The number of clusters decreases with increasing distance from the centre, *R*^2^ = .18, *F*(1,113) = 24.15, *p*_corrected_ < .001.

### Modulation of Primary Auditory Cortex during Auditory Stimulation

**Fig 6:**
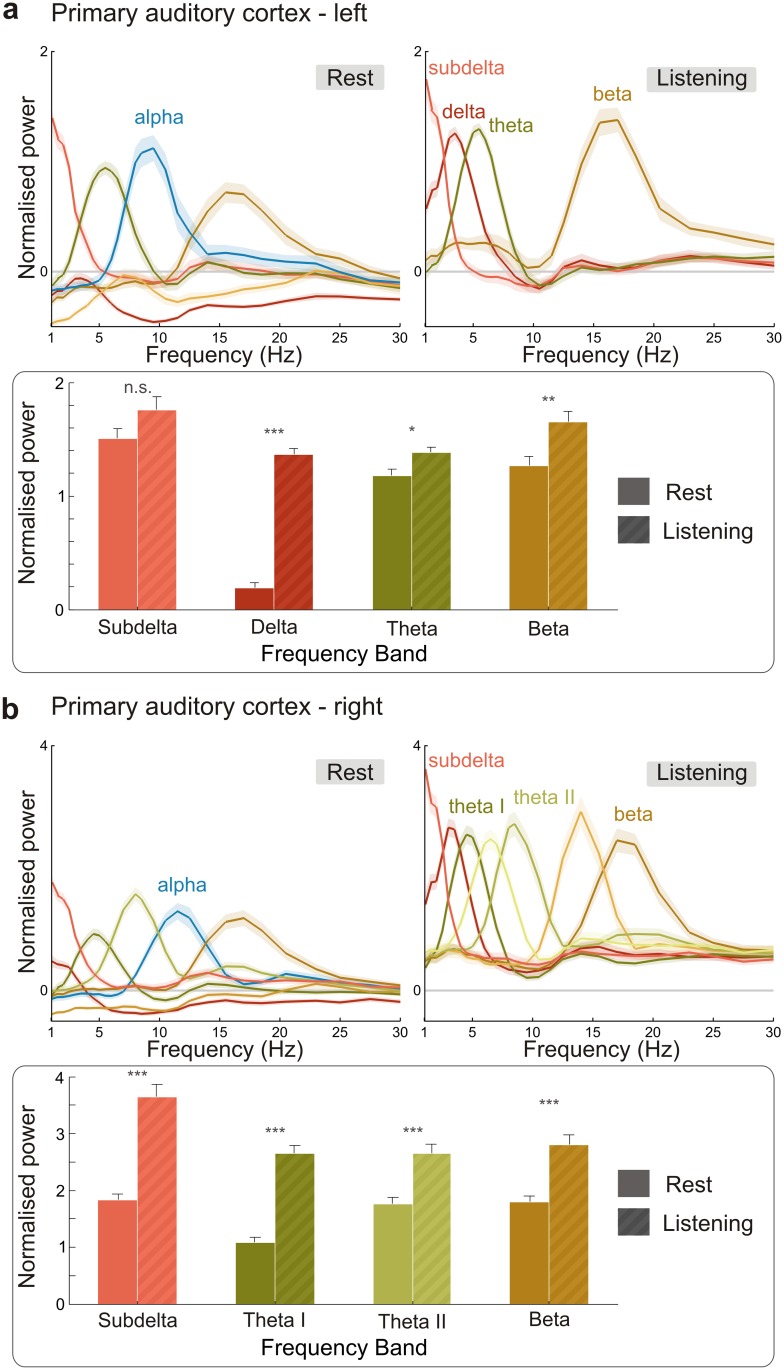
Comparison of spectral profiles in (a) left and (b) right primary auditory cortices during rest and listening. Upper panels for left and right PAC show spectral profiles including all modes for the rest (left) and listening (right) conditions. The *x*-axis is limited to 30 Hz. The amplitudes of spectra with comparable peak frequencies in both conditions were subjected to an independent sample *t* test across participants (lower panels). Bars illustrate amplitude means across individual spectra that contributed to group clusters. Error bars illustrate standard error of the mean across participants. Asterisks denote significance between rest and listening: * *p* < .05, ** *p* < .01, *** *p* < .001, n.s. not significant. Data underlying this plot can be found in S3 Data.

Next, we addressed how spectral profiles change when participants engage in a task. For this analysis, we used data from the same participants listening to a continuous narrated story and repeated the cluster analysis for these auditory stimulation data. As the primary auditory cortex (PAC) comprises a relatively small and functionally distinctive area in the AAL atlas, it is well suited for a comparison between rest and listening. Fig 6 illustrates the spectral profiles for both PACs in the two conditions. Two main results emerge from this analysis. First, the general activation of both PACs during listening is evident in the overall upward shift of profiles compared with the rest condition. This is a direct result of the normalisation procedure that expresses regional changes in relation to mean activity across the brain. Second, the transition from rest to listening is characterised by significant enhancement of low-frequency modes (especially in delta and theta frequency bands) and a disappearance of alpha clusters in both PACs.

## Discussion

### Spectral Profiles of Human Brain Areas Are Characteristic

We implemented a novel analysis pipeline that allowed the identification of anatomically defined human brain areas by means of their characteristic spectral fingerprint. Our procedure was validated on resting-state data of healthy individuals but may be generalised to any other (e.g., task-specific, patient) type of data. Specifically, we computed GM models on source-reconstructed normalised power spectra derived from short segments of MEG time series. The resulting spectral profiles were characteristic for each brain area and generalised across participants. GM models were effective in classifying test data to the correct brain areas. In a ranked order, the correct area was typically assigned in first or second place (with a mean rank of 1.4, if homologue areas were considered correct). These results suggest that brain areas, in general, show characteristic spectral profiles and this quality can be exploited to classify brain areas based on their intrinsic oscillatory activity. However, one could speculate that the clustering process might be unnecessary for classification. We examined this by setting the number of clusters to *k* = 1, thus essentially simulating a single power spectrum per area. Here, the classification process yielded a mean rank of 2.3 (as opposed to 1.8 for clustered data). This suggests that (not surprisingly) average power spectra are area-specific to some extent, but the presented clustering approach improves classification and, in many cases, reveals an area’s uniqueness.

Our novel analysis pipeline and its application to resting-state data significantly extend previous efforts to characterise oscillatory brain activity. Recent studies largely agree on the fact that alpha oscillations dominate resting-state activity in occipito-parietal brain areas, and alpha/beta oscillations are prominent in sensorimotor areas [10,21,32,33], whereas activity in other frequency bands is less prominent and more dispersed in the brain [10,22,23]. The present results add significantly to these findings by providing a model-based, comprehensive characterisation of ongoing activity in individual brain areas that is suitable for classification. Together, findings from the present study and from network studies suggest that brain areas are specialised, whilst at the same time being connected in large-scale networks.

### Brain Areas Engage in Different Functional Modes

Our results illustrate that the majority of brain areas show more than one spectrum, each characterised by a different spectral peak and duration. These spectra likely represent different states or functional modes. The modes are consistent across individuals, as we only report group clusters that were evident in the majority of participants. That means at least 73% (and an average of 93%) of participants contributed to each single mode. The duration of each individual mode quantifies the prevalence of respective oscillatory dynamics within a brain area. This is demonstrated in the visual cortex (see Fig 2 for middle occipital gyrus), where two alpha clusters were found, one with a high amplitude that was present for ~20% of the time, and one with a low amplitude that was present for ~80% of the time. As participants fixated the screen throughout data acquisition, it is expected that the continuous incoming visual information leads to suppressed alpha activity for most of the time [34] (see also S4 and S5 Figs for other parietal and occipital areas with a similar pattern). Another observation is that there is an alpha cluster in right motor areas that is virtually absent in left motor areas (see Fig 2 for precentral gyrus and S3 Fig for other central areas where this is the case). An asymmetry in central alpha amplitude has previously been observed during rest and is associated with handedness and the dominant hemisphere [35,36]. As all participants were right-handed, this alpha asymmetry in our analysis is likely reflected in the presence of right hemisphere sensorimotor alpha clusters that are largely absent in the left sensorimotor areas. However, further studies will need to address in more detail how switching of modes relates to state changes in the brain.

### Spectral Modes and Natural Frequencies

The notion of intrinsic frequency tuning, or “resonance,” is well established at the level of individual neurons [37,38] and neuronal populations [39,40]. The basic idea is that neuronal activity depends on the frequency of the input, and that (groups of) neurons have preferred “natural” frequencies where their response is enhanced. For example, Rosanova et al. [40] investigated EEG responses to single transcranial magnetic stimulation (TMS) pulses over three cortical areas. They illustrated that TMS pulses induced EEG oscillations that were specific to stimulation sites. Importantly, these natural frequencies are reflected by spectral peaks in our profiles. In detail, Rosanova et al. [40] found that the maximum power of EEG oscillations was specific to occipital (Brodmann Area 19, 11 Hz versus middle occipital gyrus, 10.5 Hz in our results), parietal (Brodmann Area 7, 20 Hz versus superior parietal gyrus, 18.5 Hz in our results), and frontal sites (Brodmann Area 6, 31 Hz versus paracentral lobule, 27.5 Hz in our results). Interestingly, the natural frequency in parietal and frontal areas was reflected by the mode with the longest duration, whereas the natural frequency in the occipital lobe was reflected by the mode with the highest amplitude. Similarly, Ferrarelli and colleagues [41] studied natural frequencies in healthy individuals and patients with schizophrenia and found specific frequencies in prefrontal, premotor, motor, and parietal cortices. These natural frequencies also correspond to peaks in the spectra of our area-specific profiles. Thus, the notion of area-specific intrinsic tuning, or natural frequencies, fits well with characteristic spectral modes in different regions of the brain.

### Areas with Similar Spectral Profiles Resemble Networks

We also analysed the similarity of spectral profiles across areas, using negative log-likelihood between areas as distance measure for hierarchical clustering. This approach yielded several clusters of brain areas that resemble resting-state brain networks often found in MEG [7,10] and fMRI [4,5,42] studies. Most prominent were clusters that resemble the sensorimotor, frontal, and visual networks (e.g., [7]). This is remarkable for two reasons: First, these well-known cortical subdivisions emerge from the clustering that is purely based on spectral profiles—i.e., the clustering algorithm has no information about the spatial location of individual brain areas. Second, this demonstrates that in spite of their uniqueness, spectral fingerprints show a graded, hierarchical similarity structure that is in agreement with basic anatomical cortical subdivisions. This is not simply an artefact of neighbouring areas showing correlated spectral profiles in the MEG, because distant homologue areas in the left and right hemispheres are clustered together, due to the similarity of their spectral profiles.

### Organisational Principles of Spectral Profiles

Regression analyses revealed systematic dependencies on an area’s location in the brain (spherical coordinates) for a number of measures (see also analysis of peak frequency in supporting information S1 Text and S12 Fig). First, we tested the “degree of uniqueness” of individual areas. The mean rank per area increases slightly with increasing radius from the centre of the brain. In principle, subcortical areas towards the middle of the brain tend to be easier to classify than areas on the cortical surface. A better classification in deeper areas cannot be caused by a higher number of clusters, as cluster numbers were kept constant across areas for the computation of GM training models. Thus, we conclude that oscillatory activity patterns of deeper areas tend to be more specific, whereas activity in areas towards the cortical surface resembles that of other cortical areas more closely.

Second, we tested the number of different functional modes that areas engage in, in relation to their location. The number of modes per area increases towards the centre of the brain. This might point towards a higher complexity in these areas. For example, all areas of the basal ganglia engage in a consistently high number of modes (optimal number of clusters between five and nine; see S8 Fig). The basal ganglia are strongly interconnected with the cerebral cortex, the thalamus, and the brainstem (e.g., [43]), and they are organised into structurally and functionally distinct “circuits” [44]. We speculate that area-specific states could reflect different modes of communication with other areas. Importantly, it is unlikely that a higher number of modes in subcortical areas is an artefact of spatial filtering or of a signal mixing of surrounding areas. Several areas of the limbic system are close to the centre of the brain but show a low number of modes (see S7 Fig). On the other hand, there are many areas on the cortical surface that show a high number of modes. Furthermore, a low signal-to-noise ratio (as in deeper brain structures) would not lead to more clusters, as spectral peaks become less distinct with increasing noise in the data. Consequently, it seems unlikely that these effects are caused by artefacts.

### Auditory Stimulation Distinctly Alters Spectral Modes in Primary Auditory Cortices

A comparison of spectral profiles in the PAC between rest and listening conditions reveals a number of interesting and important findings. First of all, the majority of modes have comparable peak frequencies, both between left and right PAC and between rest and listening. Furthermore, during listening, power amplitudes are generally enhanced, and oscillatory activity in PAC is dominated by slow frequencies (delta, theta) [45,46]. A closer look at the theta rhythm during listening reveals that there is one theta-mode in the left PAC and three in the right PAC. This theta asymmetry is in line with the asymmetric sampling hypothesis [47], which states that theta is more prominent in right auditory areas. The theta rhythm has been associated with the tracking of syllables during speech perception [45]. As the syllabic rhythm fluctuates over time, it is reasonable that neural theta band activity is not constant either. This broadband activity appears in our results as three distinct theta peaks (at 4.5, 6.5, and 8.5 Hz), indicating a fluctuation over time. These distinct peaks become broader and merge if the data are spectrally smoothed further (e.g., with a multitaper of 3 Hz). Spectral smoothing is, therefore, a way to modulate the granularity of spectral profiles. Furthermore, alpha disappears during listening in both PACs, which is consistent with a gating mechanism [34,48]. And last, a beta-mode shows significantly enhanced power during listening as compared with rest. This stronger beta-activity likely reflects enhanced top-down propagation of predictions during speech processing [49,50]. In conclusion, the spectral profiles of PACs during rest and listening reveal complex modulations that can advance our understanding of specific oscillatory mechanisms involved in speech perception.

### Relevance and Implications of Present Results

Our novel analysis pipeline reveals characteristic and untangled spectral modes of brain areas and addresses several major shortcomings of previous research. First, the analysis of oscillatory activity in source space, using MEG recordings and individual MRIs, reduces the mixing of individual source signals caused by field spread that is detrimental at the sensor level. Second, the dynamic, nonstationary nature of (oscillatory) brain activity is taken into account by clustering single-trials, as opposed to analysing average power spectra. Third, the spectral normalisation procedure introduces several advantages: it makes spectra comparable across participants, diminishes the 1/f tendency, and emphasises peaks in frequency bands other than alpha. These advantages and the simple reproducibility can make the analysis of spectral profiles a useful tool in human neuroscience. Importantly, the approach allows for the extraction of invariant characteristic spectral fingerprints that generalise across participants. This is the first demonstration, to our knowledge, that the identity of anatomically defined brain areas is reflected in spontaneous oscillatory activity. The fact that our technique results in robust low-dimensional models of regionally specific ongoing brain activity makes it a highly promising candidate for novel applications.

For example, a body of research has paved the way to modulate brain oscillations with rhythmic electromagnetic stimulation (for reviews, see, [51,52]), or sensory stimulation [53,54]. The presented peak frequencies of modes could be used to inform these neurostimulation techniques and potentially aid in testing the hypothesis that brain oscillations are causally related to cognitive processes. In addition, rhythmic neurostimulation is gaining popularity in clinical applications related to the treatment of movement disorders and mental health disorders (“oscillopathies,” [14]). Spectral profiles can provide an initial guide to the frequency bands that might be reasonably used over cortical and cerebellar sites.

As spectral profiles appear to be stable biomarkers, and brain oscillations are known to be altered in mental health disorders, this approach could be used to identify (area-specific) anomalies that link to psychiatric disorders and to further facilitate early diagnosis. An automatized analysis of resting-state data of an individual patient or a group of patients (for example, suffering from schizophrenia, epilepsy, depression, etc.) could characterise patients’ aberrant oscillatory patterns in each individual area in comparison to a norm sample and readily indicate affected areas. In the same vein, these findings could be used to model age-dependent changes in spectral profiles and relate them to cognitive decline in the ageing population.

Finally, spectral profiles may allow for a more comprehensive analysis of regionally specific modulations of brain oscillations between rest and different continuous tasks (e.g., auditory or visual stimulation). Whereas traditional analyses are typically based on the analysis of power spectra over the whole duration of the respective task, the present approach can identify task-related changes in different components of the spectral profile.

The presented analyses were performed on macroanatomical parcellations according to the AAL atlas. This atlas was chosen because it is one of the most widely used templates in neuroscientific research. However, it is to be expected that a different parcellation would result in varying spectral profiles, at least in some areas. It will be an interesting endeavour to investigate spectral profiles using different (for example, functional) and more fine-grained definitions of brain areas.

Taken together, our novel analysis pipeline leads to the identification of stable spectral fingerprints for individual brain areas that generalise across participants. This is the first demonstration, to our knowledge, of an automatic classification of anatomically defined brain areas from their ongoing spectral activity. These robust profiles pave the way for novel applications that can improve our understanding of the intricate relationship between the anatomical and functional architecture of the human brain. Spectral profiling has the potential to inform neurostimulation, aid clinical diagnosis, and lead to more precise clinical intervention.

## Materials and Methods

### Participants and Data Acquisition

Written informed consent was obtained from all participants prior to testing. The study was approved by a local ethics committee (University of Glasgow, Faculty of Information and Mathematical Sciences; approval number FIMS00733) and conducted in accordance with the Declaration of Helsinki. MEG resting-state data were acquired for 22 healthy, right-handed participants (11 female; mean age 27.2 ± 8.0 y (*M* ± *SD*), range 19–44 y) and for approximately seven min (mean duration 487 ± 22 s, range 442–521 s).

MEG-recordings were obtained with a 248-magnetometer whole-head MEG system (MAGNES 3600 WH, 4-D Neuroimaging) in a magnetically shielded room. Data were recorded at a sampling rate of 1,017 Hz. Head position was measured at the beginning and end of a run via a set of five coils that were placed on the participant’s head and that were codigitised with head-shape (FASTRAK, Polhemus Inc., VT, US). Participants sat upright and fixated on a cross projected centrally on a screen with a DLP projector. In the same session, participants listened to two 7-min long auditory stimuli. These data have been published elsewhere [46]. However, we utilise one of these conditions (participants listening to the story “Pie-man,” told by Jim O’Grady) to demonstrate the modulation of resting-state profiles during auditory processing. Auditory stimuli and rest blocks were presented in a randomised order.

### Data Preprocessing and Artefact Rejection

For MEG data analysis, we used Matlab (The MathWorks Inc), including in-house MATLAB routines and the FieldTrip toolbox [55]. The MEG signal was resampled to 250 Hz, denoised with information from the MEG reference sensors, and detrended. Noisy MEG channels were rejected when their mean z-score exceeded a value of 1.5 (mean 4.05 ± 3.24, range 1–14 channels). Continuous MEG recordings were then segmented into 1-s segments (also referred to as trials). Noisy segments were rejected when their mean z-score exceeded a value of 2. A mean of 21 segments per participant was removed (resulting in a mean of 466 ± 20, and ranging from 428 to 499 remaining segments that were included in subsequent analyses). The automatic rejection of channels and segments was visually validated. Artefacts related to heartbeats and eye blinks were removed using independent component analysis (ICA) on a 30-dimensional subspace defined by principal component analysis (PCA). We visually identified heart and eye artefacts for each participant and removed their components (mean 2.23 ± 0.43, range 2–3 components).

### Source Localisation

Individual structural magnetic resonance images (MRIs; T1-weighted) were coregistered to the MEG coordinate system by using participants’ digitised head shapes. MRIs were further realigned with participants’ individual head shapes through an iterative closest point (ICP) algorithm [56]. MRIs were then segmented to obtain a representation of the brain including white matter, grey matter, and cerebrospinal fluid. A single-shell model was used for the forward solution to construct a volume conduction model [57]. Individual anatomical MRIs were linearly transformed to a template (Montreal Neurological Institute) brain using Fieldtrip/SPM5. We computed LCMV beamformer coefficients from the MEG time series for each of the voxels on a 10-mm regular grid covering the brain. These LCMV weights were used for the later projection of complex Fourier spectra into source space. The optimal dipole orientation for each voxel was computed using the SVD approach. We used the AAL atlas [24] to parcellate the template brain into 116 anatomical areas, including a cerebellar parcellation [58]. Note that one area of the AAL atlas (Cerebellum 3L) was not interpolated with the 10-mm source model. This area was excluded from all analyses, yielding 115 areas in total.

### Spectral Analysis

Complex sensor-level Fourier-spectra for single segments were computed using the Fast Fourier Transform. The signal was zero-padded to a length of 2 s and spectrally smoothed (+/− 2 Hz) with a DPSS multitaper. We used a logarithmic frequency resolution for the spectral analysis to optimise resolution of lower frequencies, resulting in 42 frequency values between 1 and 120 Hz (6 frequencies in the delta band, 9 in the theta band, 5 in the alpha band, 8 in the beta band, and 14 in the gamma band). Complex sensor-level Fourier-spectra were projected into source space for each individual 1-s data segment by using the previously computed LCMV weights. Data were spectrally normalised by dividing the spectrum of each segment and voxel by the average power spectrum across all segments and voxels per participant (ratio normalisation). This normalisation results in values above/below one. To make an increase or decrease in power more apparent, all values were subtracted by one (resulting in values above/below zero). We then averaged single-segment normalised power spectra across voxels separately for each brain area according to the parcellation of the AAL atlas. Thus, results represent spectral activity in each brain area in comparison to the rest of the brain.

### k-Means Clustering and GM Modelling of Spectral Data

We used *k*-means clustering and subsequent GM modelling at individual and group levels to partition frequency spectra within each anatomical ROI into distinct clusters. First, single-trial power spectra of each participant were partitioned into ten clusters per anatomical area using *k*-means to reduce data dimensionality. The *k*-means algorithm treats each single segment power spectrum as a point in 42-dimensional space and groups data into *k* mutually exclusive clusters by minimising the centroid distance of observations within clusters and maximising the distance between clusters. We used a cosine distance metric, which means that one minus the cosine of the included angle between points determined the distance within and between clusters. By using a cosine distance measure, the clustering procedure focuses on the shape of spectra, as opposed to amplitudes (e.g., when using Euclidean distance). Ten individual clusters per ROI were predefined, because this yielded the best results for the subsequent clustering over participants in our data. The *k*-means algorithm was repeated 10 times with different (randomly determined) centroids and maximal 100 iterations; the solution with the lowest sum of distances within clusters was accepted. Second, GM models were fitted separately to the ten clusters of each ROI for each of the 22 participants (1st-level GM models). GM models are parametric probability density functions represented as a weighted sum of Gaussian component densities. The fitting uses an iterative expectation maximization algorithm, which assigns posterior probabilities (using negative log-likelihood) to each component density with respect to each observation. Importantly, posterior probabilities indicate the probability of each observation to belong to a cluster and therefore provide a possibility to statistically test clustering solutions.

For the clustering at group level, the optimal number of clusters per ROI was estimated using a Silhouette criterion with a cosine distance metric. The Silhouette value measures how similar a point is to other points in its own cluster, when compared to points in other clusters. The computation of Silhouette values for cluster solutions between 1–15 clusters was repeated 1,000 times. The number of clusters with the highest Silhouette value was chosen. The best *k*-means solution (lowest sum of distances out of 10 replicates with 100 iterations) was used as a starting point for the GM modelling algorithm to obtain the final set of clusters for each anatomical area (2nd-level GM models). We only accepted clusters that were representative for the majority of participants (*N* = 16, *Χ*^*2*^(1) = 4.55, *p* = .03). Furthermore, the average duration that each cluster was evident during the recording was computed. This was done by counting the number of individual trials that contributed to a specific cluster, expressing this in percent, and averaging across participants. Note that this procedure can yield cumulative percentages of above 100%.

For linear regression analyses with measures that resulted from 2nd-level GM models (such as mean rank, number of clusters, or peak frequency per area), the Cartesian coordinates of each brain area were averaged and their spherical counterparts computed. Regression was computed for radius, elevation angle, and azimuth angle, and significance levels were corrected using the sequential Bonferroni procedure [31].

### Testing the Resting-State Models of Spectral Activity

We used the following procedure to test the models of spectral activity and to determine the specificity of spectral profiles for individual areas. The 22 data sets were randomly split into training (*n* = 11) and test data (*n* = 11), and a 2nd-level GM model was computed for every brain area from the training data. Next, we computed the negative log-likelihood for every combination of area-specific test data (i.e., 1st-level GM models) and area-specific 2nd-level GM models from the training data (see Fig 3a). The negative log-likelihood quantifies the probability that the test data are consistent with the GM model. For this analysis, the number of clusters per area was set to four, which was the mode of optimal cluster numbers in the main analysis. The number of clusters was kept constant to avoid bias in negative log-likelihood that would result from a different number of clusters. Finally, for any given brain area the probabilities of the test data to fit 2nd-level GM models were ranked (e.g., test data from left precentral gyrus matched to GM models for all 115 brain areas). The classification procedure was repeated 120 times with different random assignments of training and test data. We then computed the 20% trimmed mean of ranks across all areas and iterations. A rank of 1 means the correct model area was the most likely to fit the test area; a rank of 2 means it was the second most likely, and so on.

### Similarity across Areas

The similarity across brain areas was analysed using an agglomerative hierarchical clustering procedure. Negative log-likelihood between areas of the 2nd-level GM model and data of all participants served as distance measure. The algorithm links pairs of areas depending on their proximity to each other. This procedure is repeated with each pair until all areas form a binary hierarchical cluster tree (dendrogram). We used the unweighted average distance method for computing the distance between areas and the cosine angle as metric. For the presentation of results, the cluster tree was cut at a maximum of 20 clusters.

### Comparison of Spectral Profiles during Rest and Listening

Spectral brain activity during continuous listening to a story was analysed in the same way as rest data. Spectral profiles during rest and listening were compared for primary auditory cortices (labelled “Heschl” in the AAL atlas). We focus on primary auditory cortices, as they comprise relatively small, functionally distinct areas in the AAL atlas. First, the peak of each spectrum was identified to find comparable clusters during rest and listening. The amplitude maxima of these peaks were then compared by tracing clusters back to individual 1st-level GM models. That way, at least 16 amplitude values (as each 2nd-level GM model had to consist of data from at least 16 participants) entered an independent sample *t* test, without the assumption of equal variances. All *p*-values are corrected for multiple testing using a sequential Bonferroni procedure [31].

### Code Availability

The Matlab-based script, as well as clean data and LCMV filters for all participants, can be downloaded from the MEG web site of the Centre for Cognitive Neuroimaging, Institute of Neuroscience and Psychology, University of Glasgow: http://meg.psy.gla.ac.uk/.

## Supporting Information

S1 Data Data for regression analysis of mean rank and location. (first column) Region of interest (ROI) number in AAL atlas. (second column) Mean rank of classification analysis for each area. (third column) Radius *r* (in cm) from the centre of the brain for each area (centre of ROI).
(XLSX)

S2 Data Data for regression analysis of cluster numbers and location. (first column) ROI number in AAL atlas. (second column) Optimal number of clusters for each area according to Silhouette criterion. (third column) Radius *r* (in cm) from the centre of the brain for each area (centre of ROI).
(XLSX)

S3 Data Data for comparison of spectral modes between rest and listening. (left) Tables show amplitude means and standard error of means across participants for all comparable modes in left (upper panel) and right (lower panel) PAC. (right) For the same modes, single subject data are shown. These consist of amplitudes of individual peaks that contributed to group spectra. Note that individual participants could contribute more than one value to group spectra. Sample sizes can therefore differ between conditions.
(XLSX)

S1 Fig Spectral profiles of inferior frontal areas. Clustered power spectra in source space represent normalised power, i.e., spectral power in comparison to the whole brain. Legends show the corresponding duration of each pattern (i.e., the percentage of trials in which each spectrum was present on average during recording). Shaded error bars illustrate the standard error of the mean across participants. The lines are colour-coded for the respective frequency bands (red: delta, green: theta, blue: alpha, orange: beta, grey: gamma). Inlets show average power spectra for respective areas without normalisation (dotted lines) and with ratio normalisation (continuous lines). Frequency on the *x*-axis is scaled logarithmically, and data at 50 Hz (line noise) is interpolated in the plots. Note that this division is arbitrary, and that other divisions are possible. Schematic area projections are taken from the website of the Quantitative Neuroscience Laboratory (http://qnl.bu.edu/obart/explore/AAL/) and printed with permission from Jason W. Bohland.
(TIF)

S2 Fig Spectral profiles of superior frontal areas. Clustered power spectra in source space represent normalised power, i.e., spectral power in comparison to the whole brain. Legends show the corresponding duration of each pattern (i.e., the percentage of trials in which each spectrum was present on average during recording). Shaded error bars illustrate the standard error of the mean across participants. The lines are colour-coded for the respective frequency bands (red: delta, green: theta, blue: alpha, orange: beta, grey: gamma). Inlets show average power spectra for respective areas, without normalisation (dotted lines) and with ratio normalisation (continuous lines). Frequency on the *x*-axis is scaled logarithmically, and data at 50 Hz (line noise) is interpolated in the plots. Note that this division is arbitrary and that other divisions are possible. Schematic area projections are taken from the website of the Quantitative Neuroscience Laboratory (http://qnl.bu.edu/obart/explore/AAL/) and printed with permission from Jason W. Bohland.
(TIF)

S3 Fig Spectral profiles of central areas. Clustered power spectra in source space represent normalised power, i.e., spectral power in comparison to the whole brain. Legends show the corresponding duration of each pattern (i.e., the percentage of trials in which each spectrum was present on average during recording). Shaded error bars illustrate the standard error of the mean across participants. The lines are colour-coded for the respective frequency bands (red: delta, green: theta, blue: alpha, orange: beta, grey: gamma). Inlets show average power spectra for respective areas without normalisation (dotted lines) and with ratio normalisation (continuous lines). Frequency on the *x*-axis is scaled logarithmically, and data at 50 Hz (line noise) is interpolated in the plots. Note that this division is arbitrary, and that other divisions are possible. Schematic area projections are taken from the website of the Quantitative Neuroscience Laboratory (http://qnl.bu.edu/obart/explore/AAL/) and printed with permission from Jason W. Bohland.
(TIF)

S4 Fig Spectral profiles of parietal areas. Clustered power spectra in source space represent normalised power, i.e., spectral power in comparison to the whole brain. Legends show the corresponding duration of each pattern (i.e., the percentage of trials in which each spectrum was present on average during recording). Shaded error bars illustrate the standard error of the mean across participants. The lines are colour-coded for the respective frequency bands (red: delta, green: theta, blue: alpha, orange: beta, grey: gamma). Inlets show average power spectra for respective areas, without normalisation (dotted lines) and with ratio normalisation (continuous lines). Frequency on the *x*-axis is scaled logarithmically, and data at 50 Hz (line noise) is interpolated in the plots. Note that this division is arbitrary and that other divisions are possible. Schematic area projections are taken from the website of the Quantitative Neuroscience Laboratory (http://qnl.bu.edu/obart/explore/AAL/) and printed with permission from Jason W. Bohland.
(TIF)

S5 Fig Spectral profiles of occipital areas. Clustered power spectra in source space represent normalised power, i.e., spectral power in comparison to the whole brain. Legends show the corresponding duration of each pattern (i.e., the percentage of trials in which each spectrum was present on average during recording). Shaded error bars illustrate the standard error of the mean across participants. The lines are colour-coded for the respective frequency bands (red: delta, green: theta, blue: alpha, orange: beta, grey: gamma). Inlets show average power spectra for respective areas, without normalisation (dotted lines) and with ratio normalisation (continuous lines). Frequency on the *x*-axis is scaled logarithmically, and data at 50 Hz (line noise) is interpolated in the plots. Note that this division is arbitrary and that other divisions are possible. Schematic area projections are taken from the website of the Quantitative Neuroscience Laboratory (http://qnl.bu.edu/obart/explore/AAL/) and printed with permission from Jason W. Bohland.
(TIF)

S6 Fig Spectral profiles of temporal areas. Note that this division is arbitrary, and that other divisions are possible. Clustered power spectra in source space represent normalised power, i.e., spectral power in comparison to the whole brain. Legends show the corresponding duration of each pattern (i.e., the percentage of trials in which each spectrum was present on average during recording). Shaded error bars illustrate the standard error of the mean across participants. The lines are colour-coded for the respective frequency bands (red: delta, green: theta, blue: alpha, orange: beta, grey: gamma). Inlets show average power spectra for respective areas without normalisation (dotted lines) and with ratio normalisation (continuous lines). Frequency on the *x*-axis is scaled logarithmically, and data at 50 Hz (line noise) is interpolated in the plots. Note that this division is arbitrary, and that other divisions are possible. Schematic area projections are taken from the web site of the Quantitative Neuroscience Laboratory (http://qnl.bu.edu/obart/explore/AAL/) and printed with permission from Jason W. Bohland.
(TIF)

S7 Fig Spectral profiles of areas of the limbic system. Clustered power spectra in source space represent normalised power, i.e., spectral power in comparison to the whole brain. Legends show the corresponding duration of each pattern (i.e., the percentage of trials in which each spectrum was present on average during recording). Shaded error bars illustrate the standard error of the mean across participants. The lines are colour-coded for the respective frequency bands (red: delta, green: theta, blue: alpha, orange: beta, grey: gamma). Inlets show average power spectra for respective areas, without normalisation (dotted lines) and with ratio normalisation (continuous lines). Frequency on the *x*-axis is scaled logarithmically, and data at 50 Hz (line noise) is interpolated in the plots. Note that this division is arbitrary and that other divisions are possible. Schematic area projections are taken from the website of the Quantitative Neuroscience Laboratory (http://qnl.bu.edu/obart/explore/AAL/) and printed with permission from Jason W. Bohland.
(TIF)

S8 Fig Spectral profiles of areas of the basal ganglia. Clustered power spectra in source space represent normalised power, i.e., spectral power in comparison to the whole brain. Legends show the corresponding duration of each pattern (i.e., the percentage of trials in which each spectrum was present on average during recording). Shaded error bars illustrate the standard error of the mean across participants. The lines are colour-coded for the respective frequency bands (red: delta, green: theta, blue: alpha, orange: beta, grey: gamma). Inlets show average power spectra for respective areas without normalisation (dotted lines) and with ratio normalisation (continuous lines). Frequency on the *x*-axis is scaled logarithmically, and data at 50 Hz (line noise) is interpolated in the plots. Note that this division is arbitrary and that other divisions are possible. Schematic area projections are taken from the website of the Quantitative Neuroscience Laboratory (http://qnl.bu.edu/obart/explore/AAL/) and printed with permission from Jason W. Bohland.
(TIF)

S9 Fig Spectral profiles of areas of the cerebellum. Clustered power spectra in source space represent normalised power, i.e., spectral power in comparison to the whole brain. Legends show the corresponding duration of each pattern (i.e., the percentage of trials in which each spectrum was present on average during recording). Shaded error bars illustrate the standard error of the mean across participants. The lines are colour-coded for the respective frequency bands (red: delta, green: theta, blue: alpha, orange: beta, grey: gamma). Inlets show average power spectra for respective areas without normalisation (dotted lines) and with ratio normalisation (continuous lines). Frequency on the *x*-axis is scaled logarithmically, and data at 50 Hz (line noise) is interpolated in the plots. Note that this division is arbitrary and that other divisions are possible. Schematic area projections are taken from the website of the Quantitative Neuroscience Laboratory (http://qnl.bu.edu/obart/explore/AAL/) and printed with permission from Jason W. Bohland.
(TIF)

S10 Fig Spectral profiles of areas of the cerebellar vermis. Clustered power spectra in source space represent normalised power, i.e., spectral power in comparison to the whole brain. Legends show the corresponding duration of each pattern (i.e., the percentage of trials in which each spectrum was present on average during recording). Shaded error bars illustrate the standard error of the mean across participants. The lines are colour-coded for the respective frequency bands (red: delta, green: theta, blue: alpha, orange: beta, grey: gamma). Inlets show average power spectra for respective areas, without normalisation (dotted lines) and with ratio normalisation (continuous lines). Frequency on the *x*-axis is scaled logarithmically, and data at 50 Hz (line noise) is interpolated in the plots. Note that this division is arbitrary and that other divisions are possible. Schematic area projections are taken from the website of the Quantitative Neuroscience Laboratory (http://qnl.bu.edu/obart/explore/AAL/) and printed with permission from Jason W. Bohland.
(TIF)

S11 Fig Binary hierarchical cluster tree resulting from similarity analysis of posterior probabilities. (a) Full dendrogram, cut at a maximum of 20 clusters. (b and c) Left and right part of the dendrogram with area labels.
(TIF)

S12 Fig Distribution of peak frequencies. (left) Histogram of peak frequencies with strongest power across all 115 brain areas. Bin width is adapted for each frequency band according to number of frequencies within each band. (middle) Topography of peak frequency in cluster with strongest power (colour-coded; red: delta, green: theta, blue: alpha, orange: beta, grey: gamma; bin width differs for each frequency band). (right) Linear regression of peak frequency per area and the radius *r* from the centre of the brain. The peak frequency decreases the closer an area is to the centre of the brain. Areas in the middle of the brain mostly show peaks in the delta band, whereas higher peak frequencies were found in areas further away from the centre.
(TIF)

S1 Text Peak frequency per anatomical area. Analysis of peak frequencies with strongest power for all brain areas.
(DOCX)

## Abbreviations

AAL: Automated Anatomical Labeling
EEG: electroencephalography
GM: Gaussian mixture
ICA: independent component analysis
ICP: iterative closest point
LCMV: linear constraint minimum variance
MEG: magnetoencephalography
PAC: primary auditory cortex
PCA: principal component analysis
ROI: region of interest
TMS: transcranial magnetic stimulation
