# Supplementary figures and images for: Individual Human Brain Areas Can Be Identified from Their Characteristic Spectral Activation Fingerprints
Source: PLoS Biol. 2016 Jun 29;14(6):e1002498. doi: 10.1371/journal.pbio.1002498 (PMC4927181; doi:10.1371/journal.pbio.1002498)

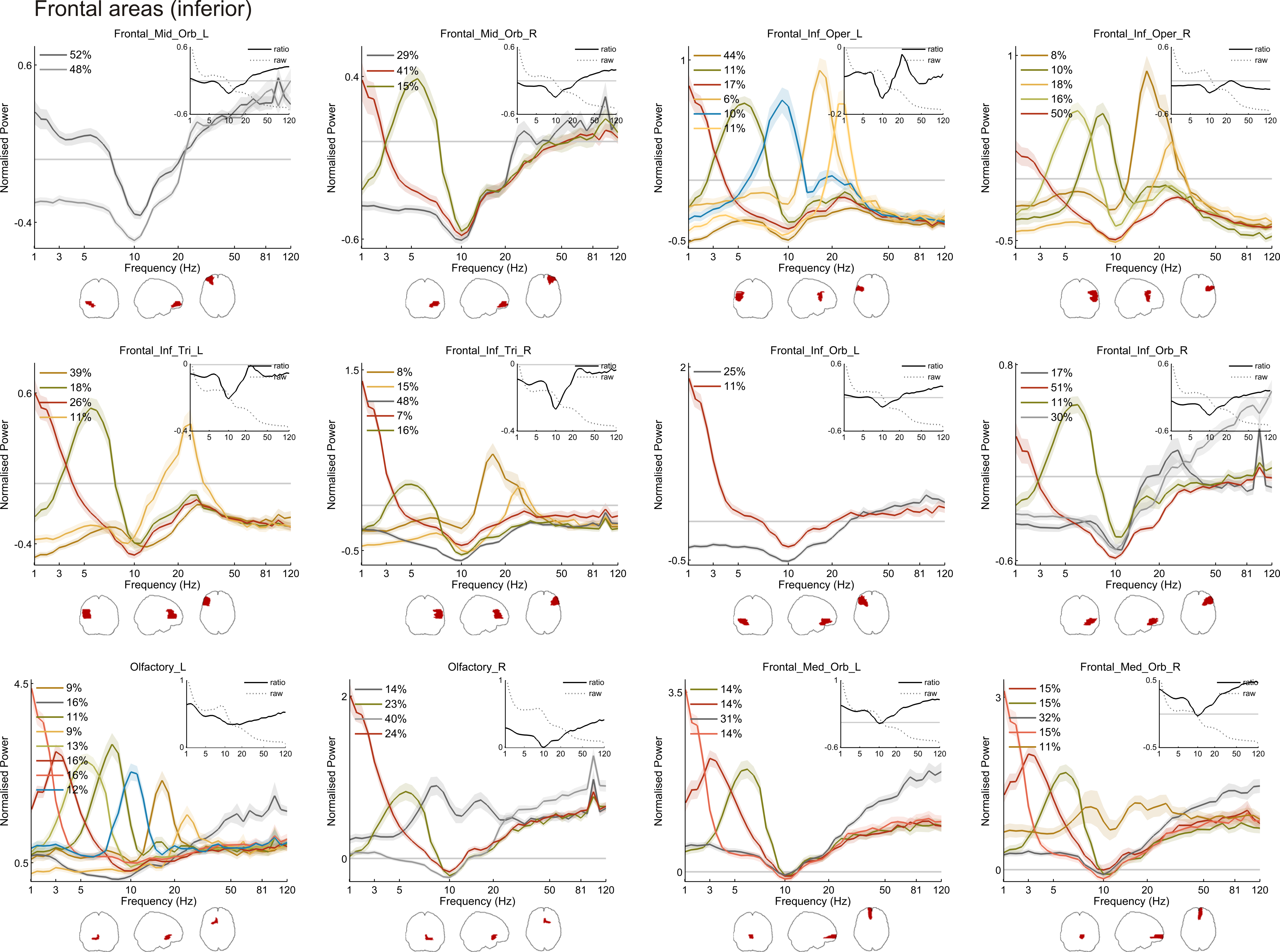

Supplement: S1 Fig — Clustered power spectra in source space represent normalised power, i.e., spectral power in comparison to the whole brain. Legends show the corresponding duration of each pattern (i.e., the percentage of trials in which each spectrum was present on average during recording). Shaded error bars illustrate the standard error of the mean across participants. The lines are colour-coded for the respective frequency bands (red: delta, green: theta, blue: alpha, orange: beta, grey: gamma). Inlets show average power spectra for respective areas without normalisation (dotted lines) and with ratio normalisation (continuous lines). Frequency on the x-axis is scaled logarithmically, and data at 50 Hz (line noise) is interpolated in the plots. Note that this division is arbitrary, and that other divisions are possible. Schematic area projections are taken from the website of the Quantitative Neuroscience Laboratory (http://qnl.bu.edu/obart/explore/AAL/) and printed with permission from Jason W. Bohland. (TIF) [file pbio.1002498.s004.tif]

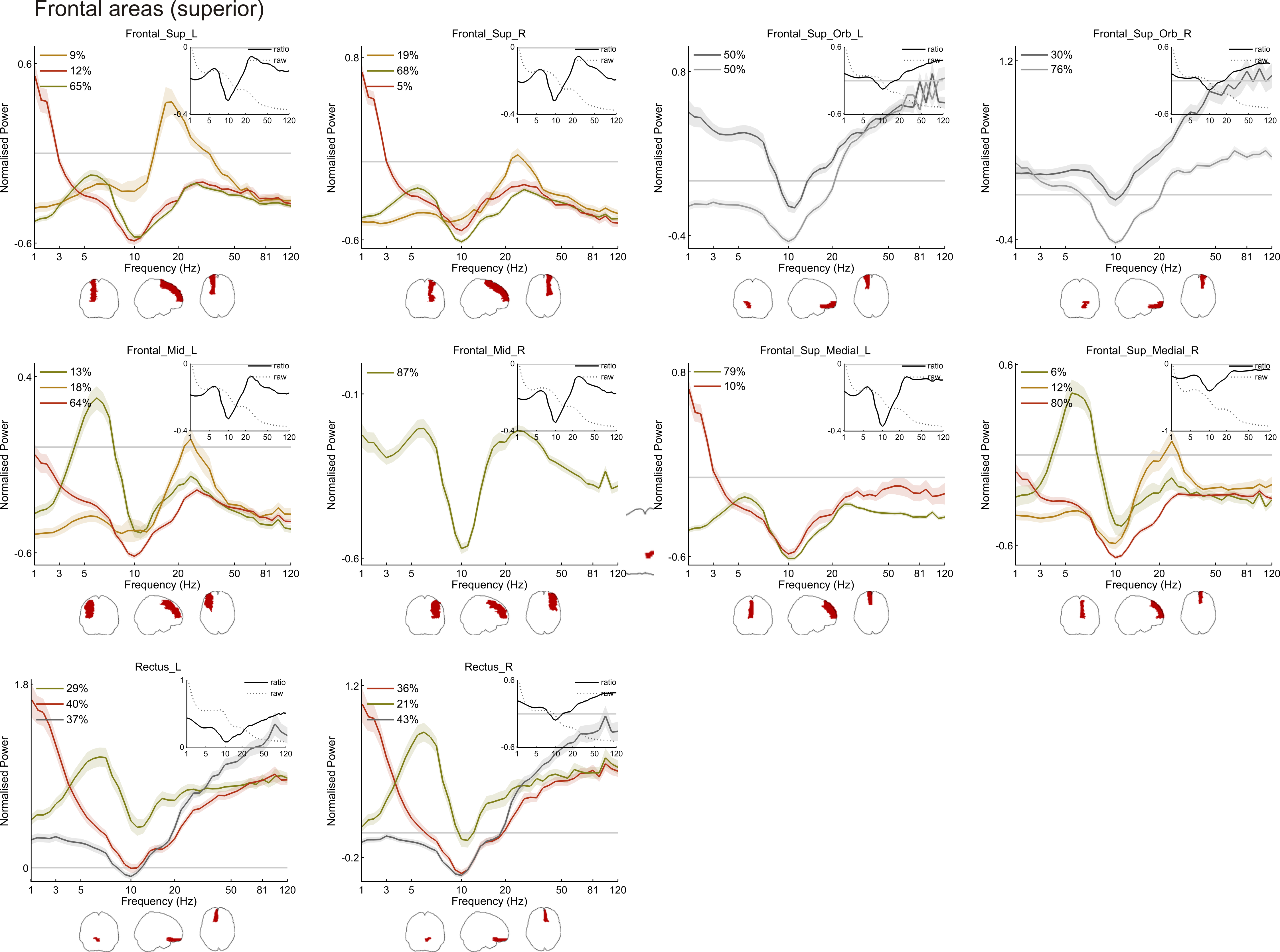

Supplement: S2 Fig — Clustered power spectra in source space represent normalised power, i.e., spectral power in comparison to the whole brain. Legends show the corresponding duration of each pattern (i.e., the percentage of trials in which each spectrum was present on average during recording). Shaded error bars illustrate the standard error of the mean across participants. The lines are colour-coded for the respective frequency bands (red: delta, green: theta, blue: alpha, orange: beta, grey: gamma). Inlets show average power spectra for respective areas, without normalisation (dotted lines) and with ratio normalisation (continuous lines). Frequency on the x-axis is scaled logarithmically, and data at 50 Hz (line noise) is interpolated in the plots. Note that this division is arbitrary and that other divisions are possible. Schematic area projections are taken from the website of the Quantitative Neuroscience Laboratory (http://qnl.bu.edu/obart/explore/AAL/) and printed with permission from Jason W. Bohland. (TIF) [file pbio.1002498.s005.tif]

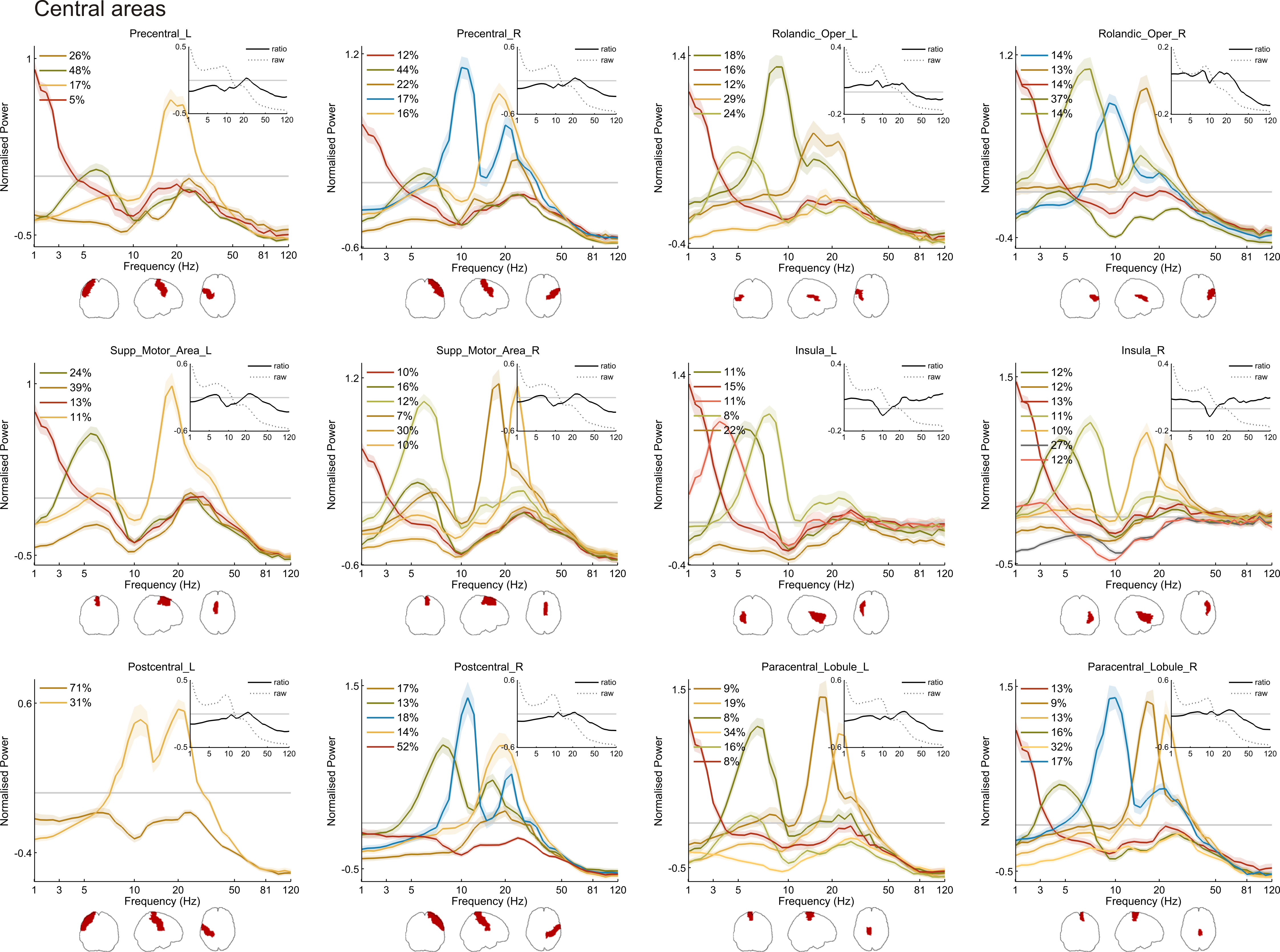

Supplement: S3 Fig — Clustered power spectra in source space represent normalised power, i.e., spectral power in comparison to the whole brain. Legends show the corresponding duration of each pattern (i.e., the percentage of trials in which each spectrum was present on average during recording). Shaded error bars illustrate the standard error of the mean across participants. The lines are colour-coded for the respective frequency bands (red: delta, green: theta, blue: alpha, orange: beta, grey: gamma). Inlets show average power spectra for respective areas without normalisation (dotted lines) and with ratio normalisation (continuous lines). Frequency on the x-axis is scaled logarithmically, and data at 50 Hz (line noise) is interpolated in the plots. Note that this division is arbitrary, and that other divisions are possible. Schematic area projections are taken from the website of the Quantitative Neuroscience Laboratory (http://qnl.bu.edu/obart/explore/AAL/) and printed with permission from Jason W. Bohland. (TIF) [file pbio.1002498.s006.tif]

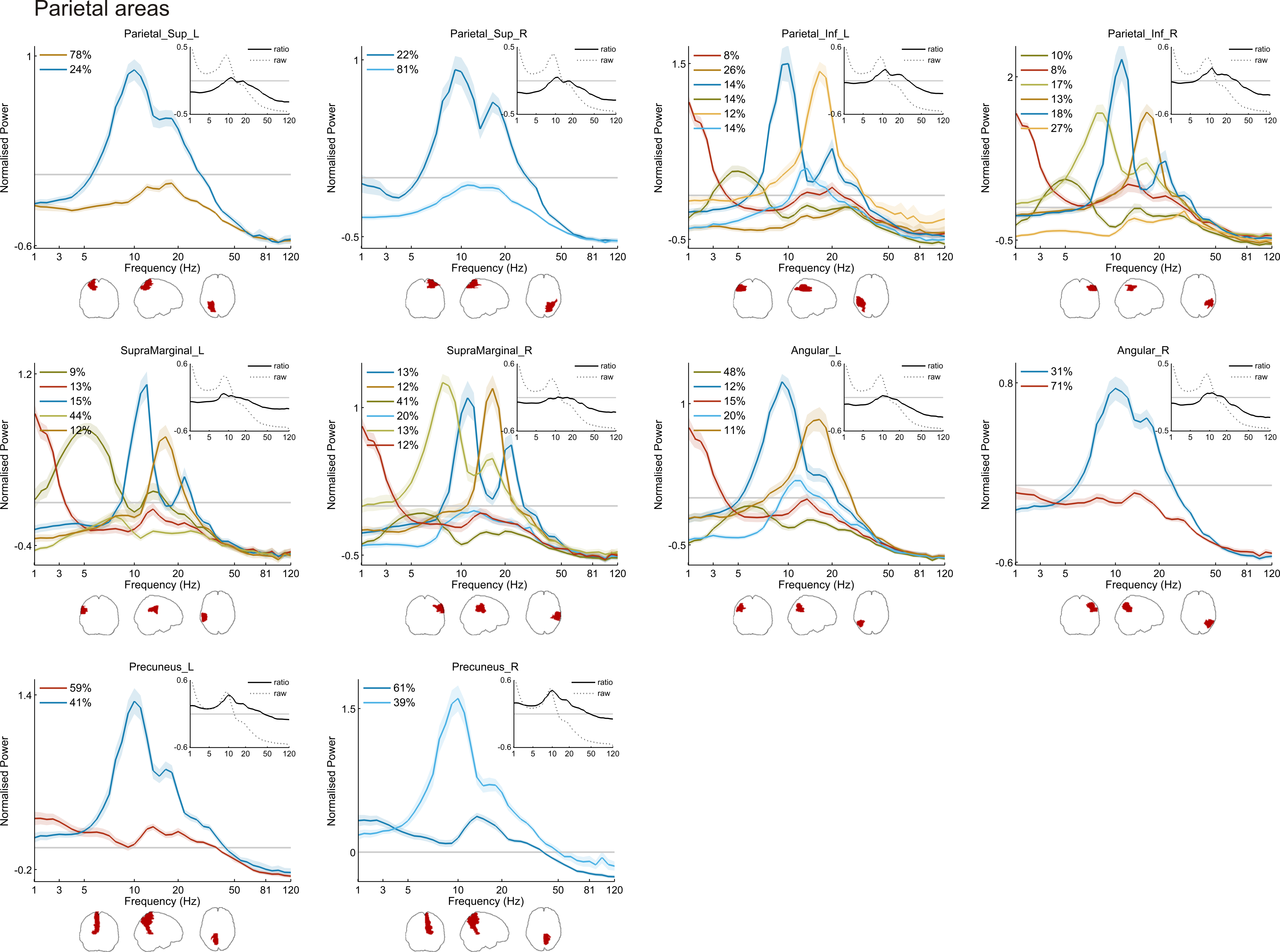

Supplement: S4 Fig — Clustered power spectra in source space represent normalised power, i.e., spectral power in comparison to the whole brain. Legends show the corresponding duration of each pattern (i.e., the percentage of trials in which each spectrum was present on average during recording). Shaded error bars illustrate the standard error of the mean across participants. The lines are colour-coded for the respective frequency bands (red: delta, green: theta, blue: alpha, orange: beta, grey: gamma). Inlets show average power spectra for respective areas, without normalisation (dotted lines) and with ratio normalisation (continuous lines). Frequency on the x-axis is scaled logarithmically, and data at 50 Hz (line noise) is interpolated in the plots. Note that this division is arbitrary and that other divisions are possible. Schematic area projections are taken from the website of the Quantitative Neuroscience Laboratory (http://qnl.bu.edu/obart/explore/AAL/) and printed with permission from Jason W. Bohland. (TIF) [file pbio.1002498.s007.tif]

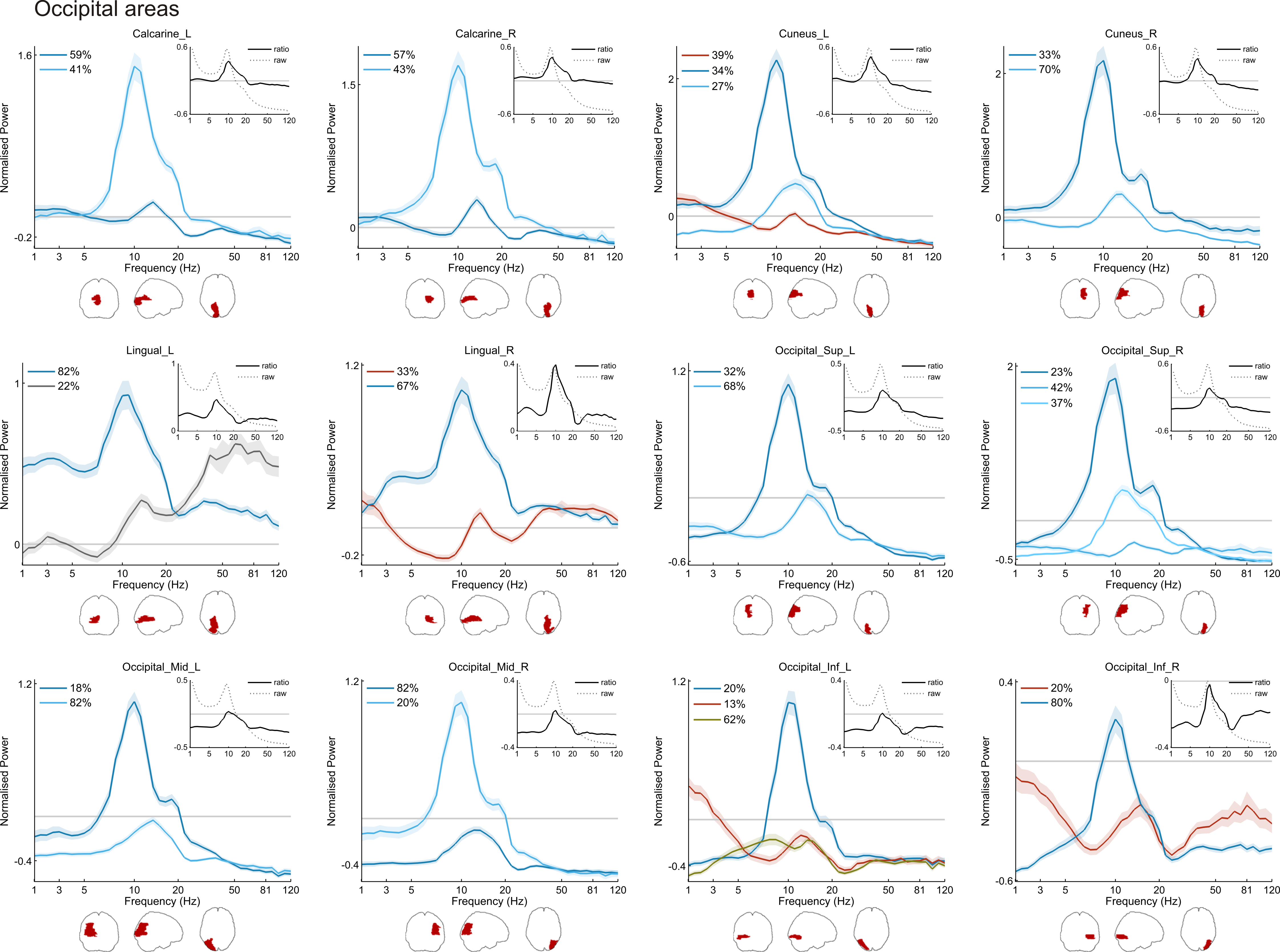

Supplement: S5 Fig — Clustered power spectra in source space represent normalised power, i.e., spectral power in comparison to the whole brain. Legends show the corresponding duration of each pattern (i.e., the percentage of trials in which each spectrum was present on average during recording). Shaded error bars illustrate the standard error of the mean across participants. The lines are colour-coded for the respective frequency bands (red: delta, green: theta, blue: alpha, orange: beta, grey: gamma). Inlets show average power spectra for respective areas, without normalisation (dotted lines) and with ratio normalisation (continuous lines). Frequency on the x-axis is scaled logarithmically, and data at 50 Hz (line noise) is interpolated in the plots. Note that this division is arbitrary and that other divisions are possible. Schematic area projections are taken from the website of the Quantitative Neuroscience Laboratory (http://qnl.bu.edu/obart/explore/AAL/) and printed with permission from Jason W. Bohland. (TIF) [file pbio.1002498.s008.tif]

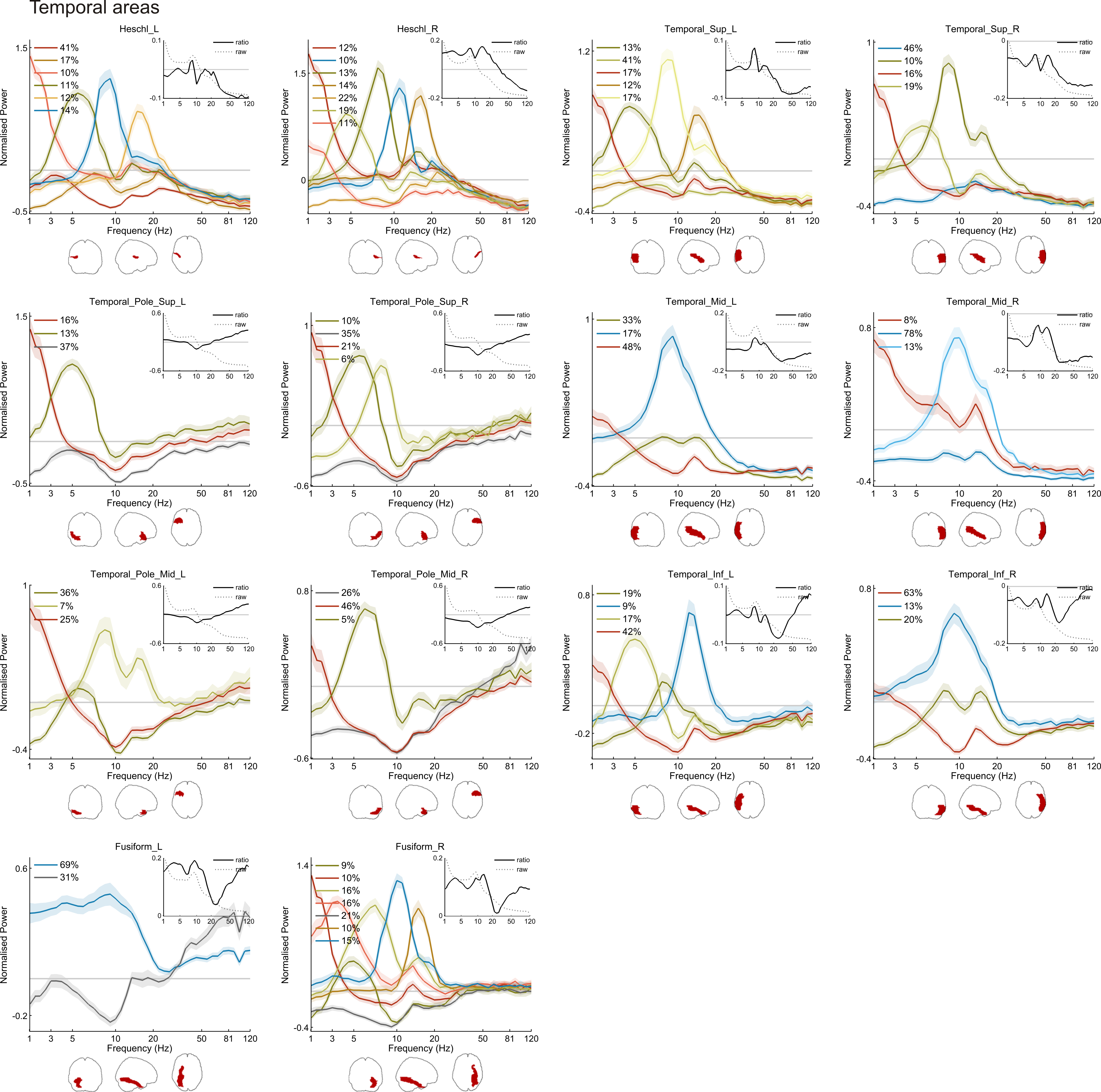

Supplement: S6 Fig — Note that this division is arbitrary, and that other divisions are possible. Clustered power spectra in source space represent normalised power, i.e., spectral power in comparison to the whole brain. Legends show the corresponding duration of each pattern (i.e., the percentage of trials in which each spectrum was present on average during recording). Shaded error bars illustrate the standard error of the mean across participants. The lines are colour-coded for the respective frequency bands (red: delta, green: theta, blue: alpha, orange: beta, grey: gamma). Inlets show average power spectra for respective areas without normalisation (dotted lines) and with ratio normalisation (continuous lines). Frequency on the x-axis is scaled logarithmically, and data at 50 Hz (line noise) is interpolated in the plots. Note that this division is arbitrary, and that other divisions are possible. Schematic area projections are taken from the web site of the Quantitative Neuroscience Laboratory (http://qnl.bu.edu/obart/explore/AAL/) and printed with permission from Jason W. Bohland. (TIF) [file pbio.1002498.s009.tif]

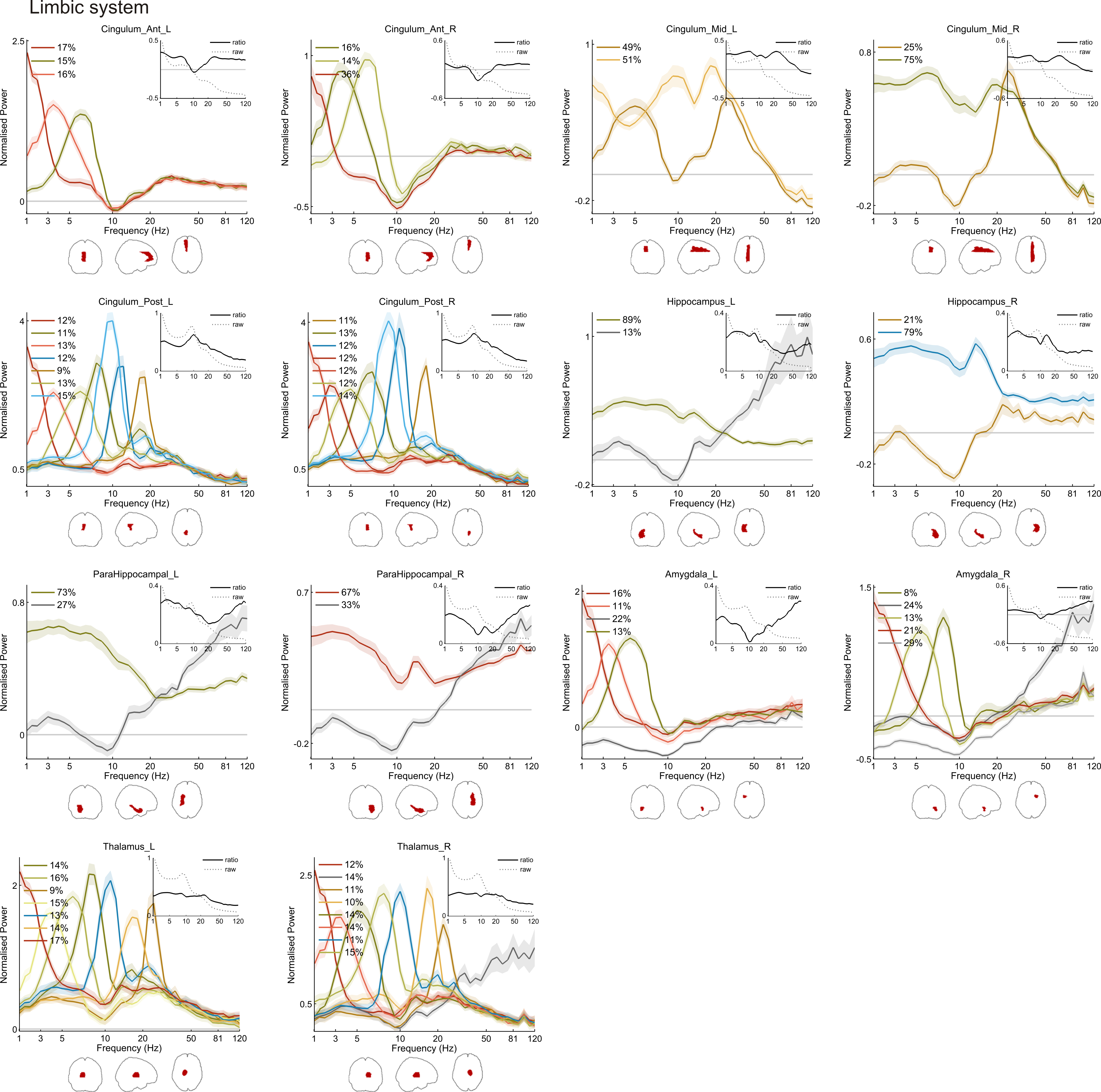

Supplement: S7 Fig — Clustered power spectra in source space represent normalised power, i.e., spectral power in comparison to the whole brain. Legends show the corresponding duration of each pattern (i.e., the percentage of trials in which each spectrum was present on average during recording). Shaded error bars illustrate the standard error of the mean across participants. The lines are colour-coded for the respective frequency bands (red: delta, green: theta, blue: alpha, orange: beta, grey: gamma). Inlets show average power spectra for respective areas, without normalisation (dotted lines) and with ratio normalisation (continuous lines). Frequency on the x-axis is scaled logarithmically, and data at 50 Hz (line noise) is interpolated in the plots. Note that this division is arbitrary and that other divisions are possible. Schematic area projections are taken from the website of the Quantitative Neuroscience Laboratory (http://qnl.bu.edu/obart/explore/AAL/) and printed with permission from Jason W. Bohland. (TIF) [file pbio.1002498.s010.tif]

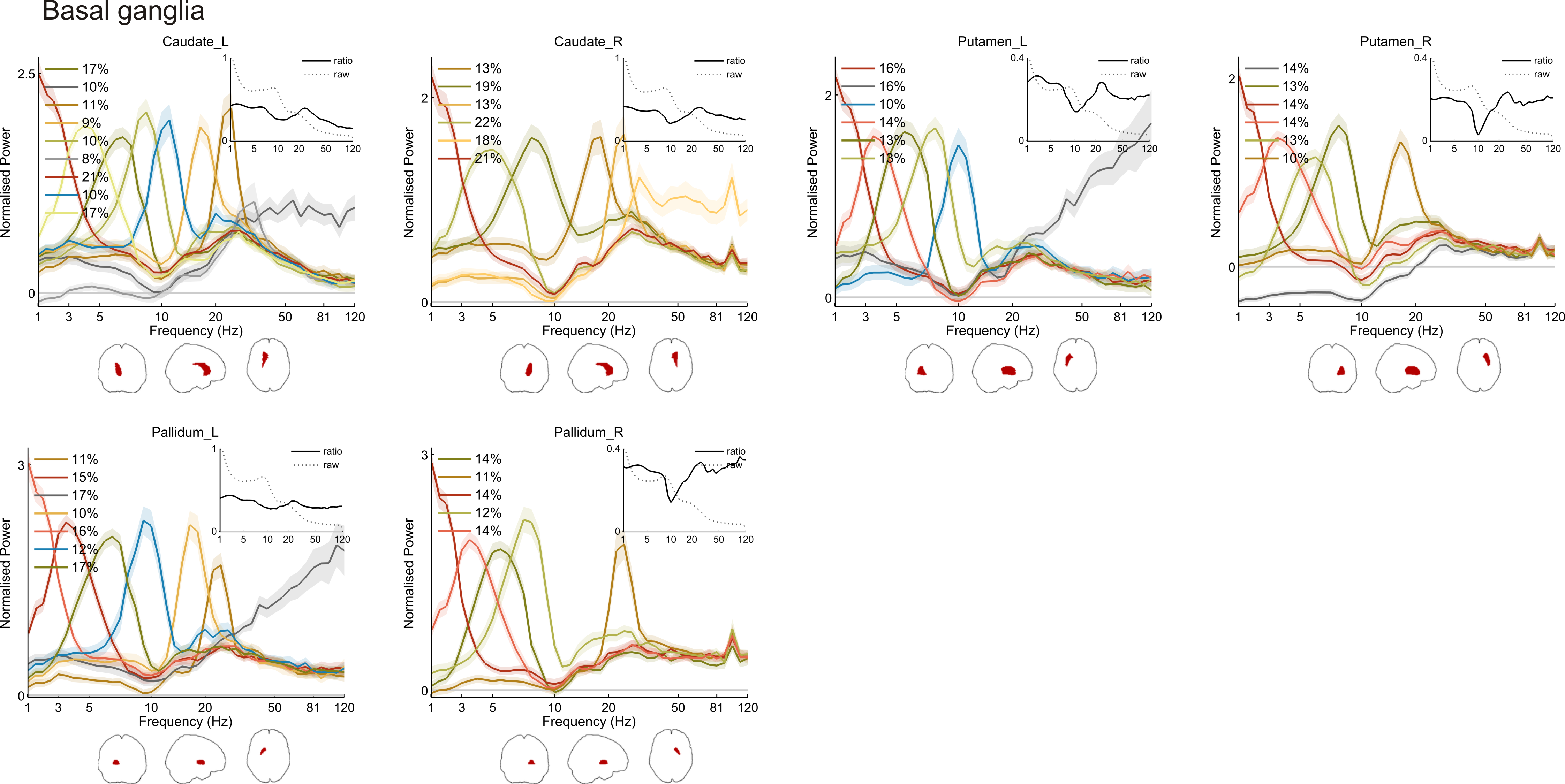

Supplement: S8 Fig — Clustered power spectra in source space represent normalised power, i.e., spectral power in comparison to the whole brain. Legends show the corresponding duration of each pattern (i.e., the percentage of trials in which each spectrum was present on average during recording). Shaded error bars illustrate the standard error of the mean across participants. The lines are colour-coded for the respective frequency bands (red: delta, green: theta, blue: alpha, orange: beta, grey: gamma). Inlets show average power spectra for respective areas without normalisation (dotted lines) and with ratio normalisation (continuous lines). Frequency on the x-axis is scaled logarithmically, and data at 50 Hz (line noise) is interpolated in the plots. Note that this division is arbitrary and that other divisions are possible. Schematic area projections are taken from the website of the Quantitative Neuroscience Laboratory (http://qnl.bu.edu/obart/explore/AAL/) and printed with permission from Jason W. Bohland. (TIF) [file pbio.1002498.s011.tif]

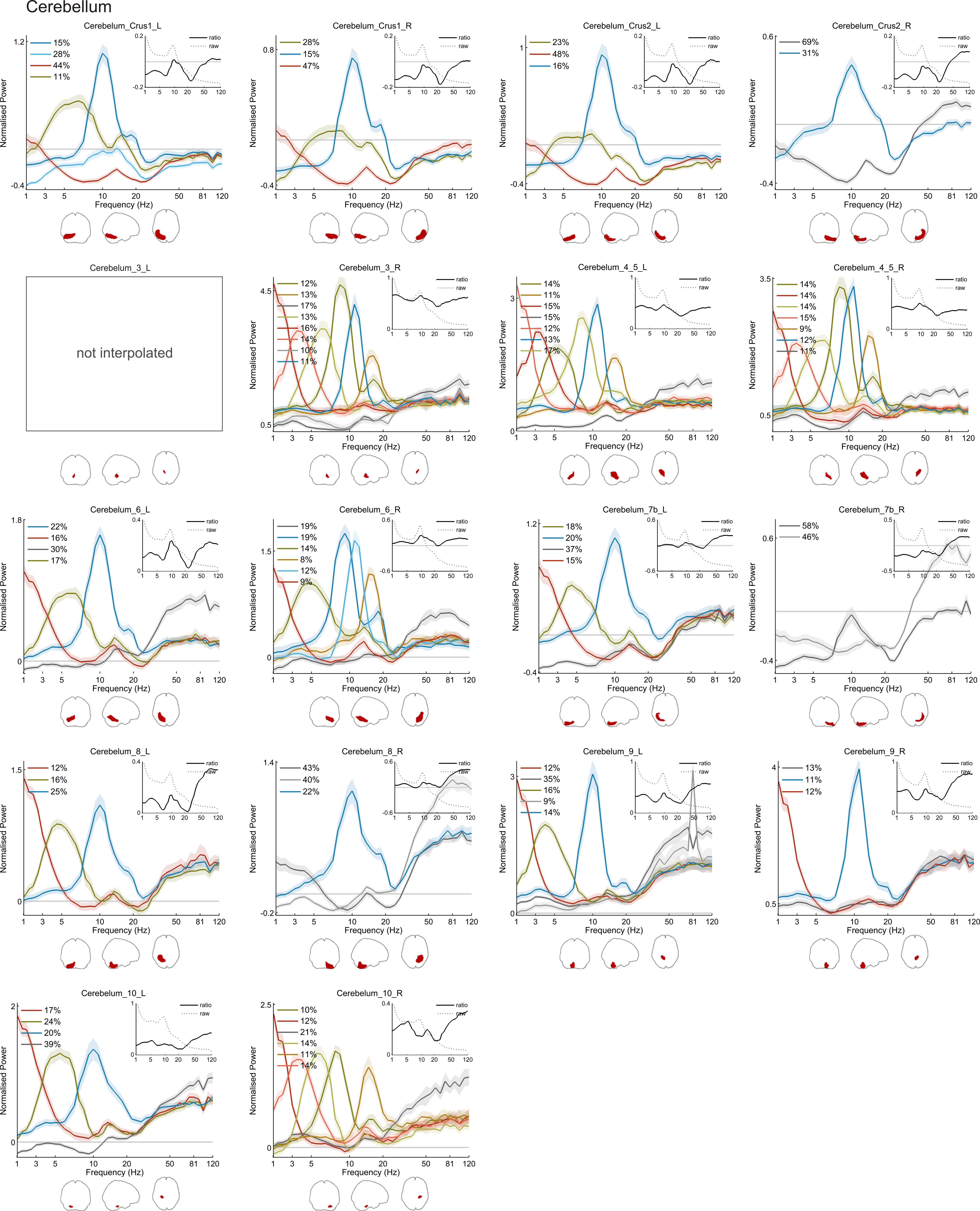

Supplement: S9 Fig — Clustered power spectra in source space represent normalised power, i.e., spectral power in comparison to the whole brain. Legends show the corresponding duration of each pattern (i.e., the percentage of trials in which each spectrum was present on average during recording). Shaded error bars illustrate the standard error of the mean across participants. The lines are colour-coded for the respective frequency bands (red: delta, green: theta, blue: alpha, orange: beta, grey: gamma). Inlets show average power spectra for respective areas without normalisation (dotted lines) and with ratio normalisation (continuous lines). Frequency on the x-axis is scaled logarithmically, and data at 50 Hz (line noise) is interpolated in the plots. Note that this division is arbitrary and that other divisions are possible. Schematic area projections are taken from the website of the Quantitative Neuroscience Laboratory (http://qnl.bu.edu/obart/explore/AAL/) and printed with permission from Jason W. Bohland. (TIF) [file pbio.1002498.s012.tif]

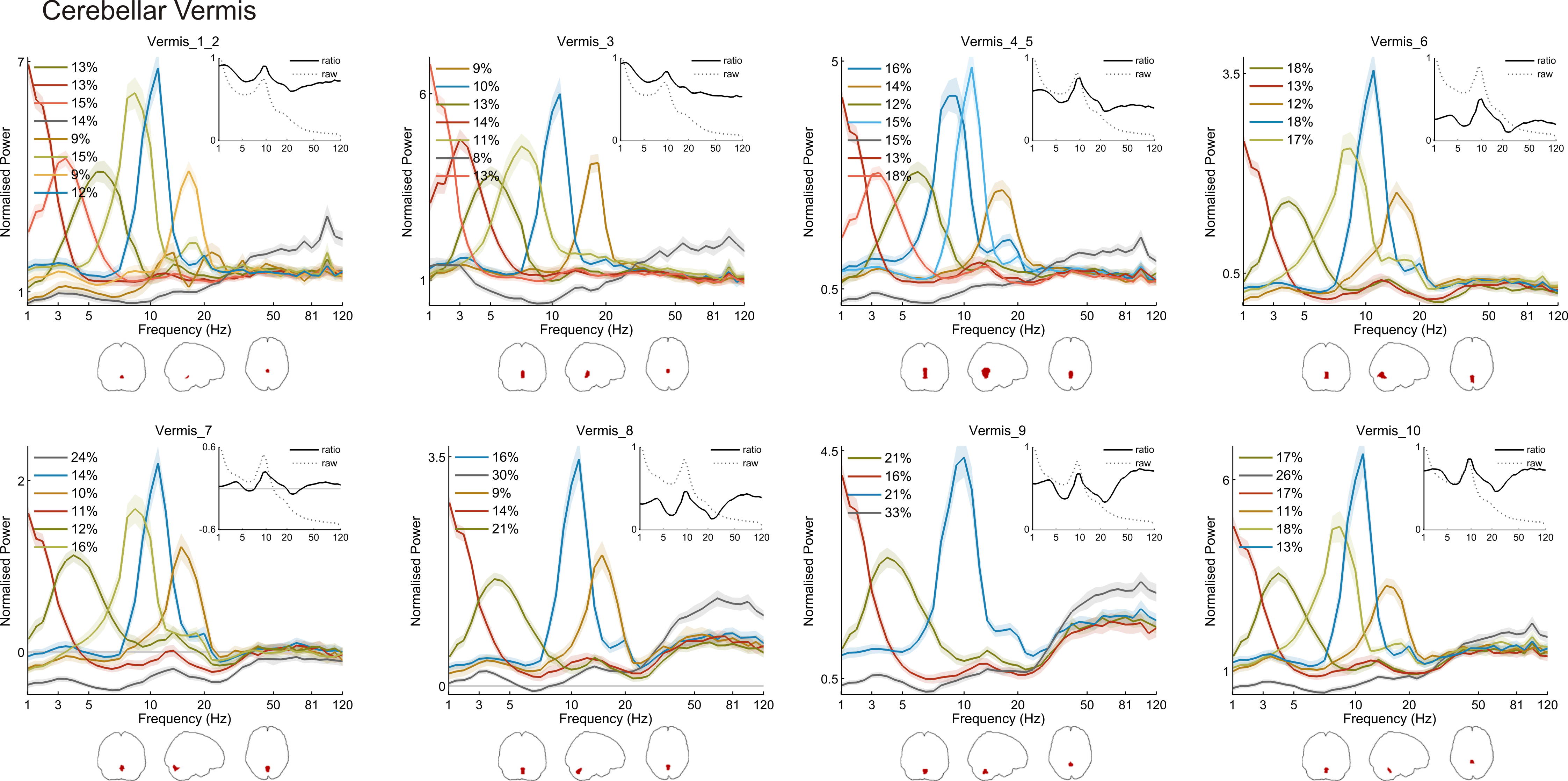

Supplement: S10 Fig — Clustered power spectra in source space represent normalised power, i.e., spectral power in comparison to the whole brain. Legends show the corresponding duration of each pattern (i.e., the percentage of trials in which each spectrum was present on average during recording). Shaded error bars illustrate the standard error of the mean across participants. The lines are colour-coded for the respective frequency bands (red: delta, green: theta, blue: alpha, orange: beta, grey: gamma). Inlets show average power spectra for respective areas, without normalisation (dotted lines) and with ratio normalisation (continuous lines). Frequency on the x-axis is scaled logarithmically, and data at 50 Hz (line noise) is interpolated in the plots. Note that this division is arbitrary and that other divisions are possible. Schematic area projections are taken from the website of the Quantitative Neuroscience Laboratory (http://qnl.bu.edu/obart/explore/AAL/) and printed with permission from Jason W. Bohland. (TIF) [file pbio.1002498.s013.tif]

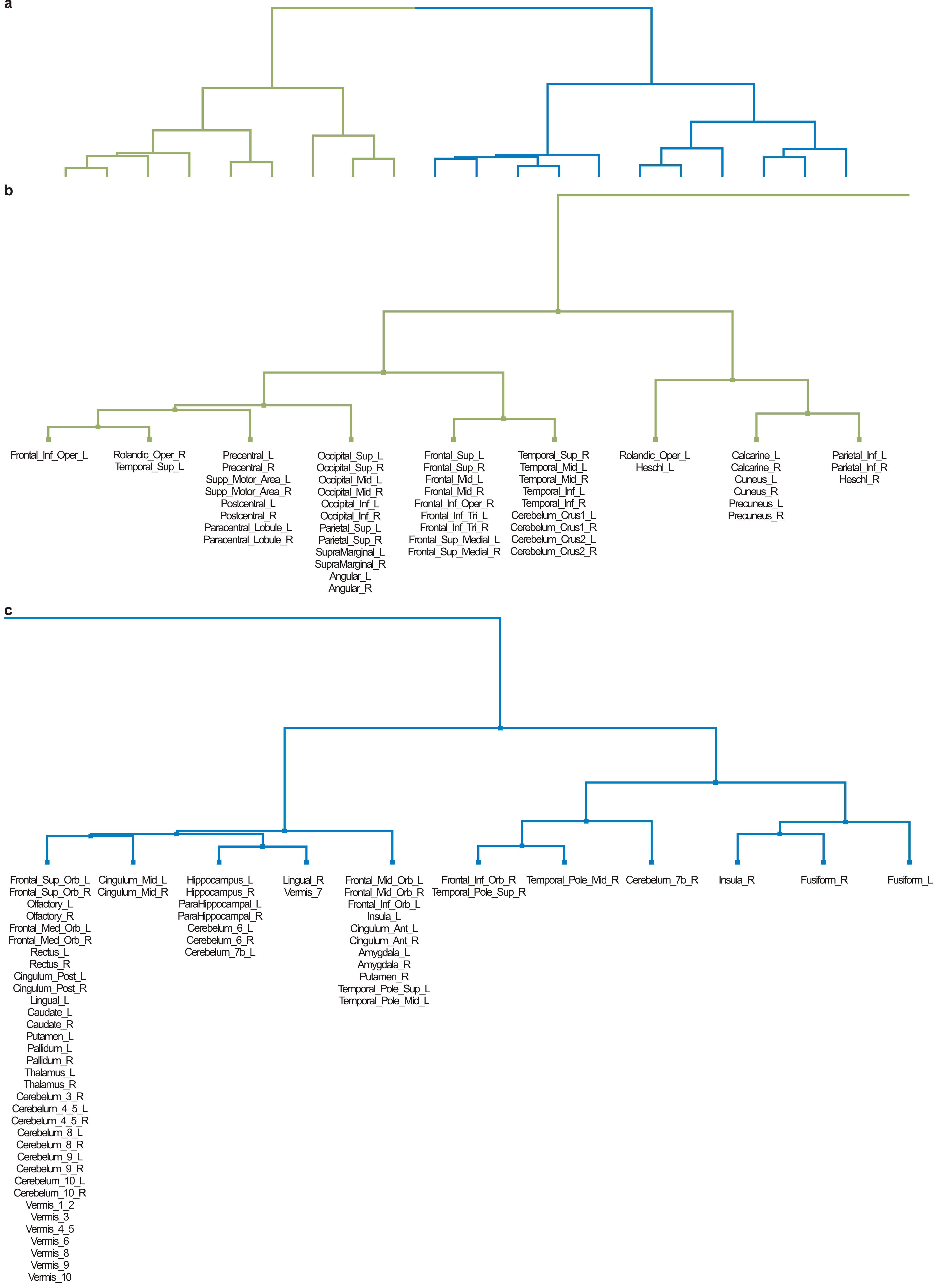

Supplement: S11 Fig — (a) Full dendrogram, cut at a maximum of 20 clusters. (b and c) Left and right part of the dendrogram with area labels. (TIF) [file pbio.1002498.s014.tif]

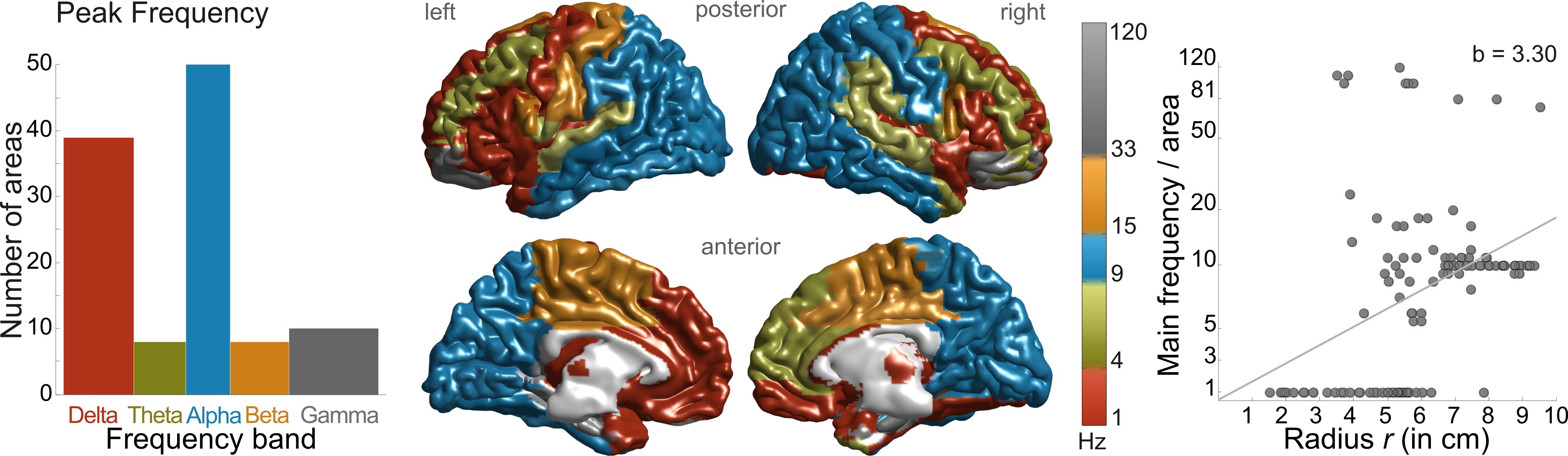

Supplement: S12 Fig — (left) Histogram of peak frequencies with strongest power across all 115 brain areas. Bin width is adapted for each frequency band according to number of frequencies within each band. (middle) Topography of peak frequency in cluster with strongest power (colour-coded; red: delta, green: theta, blue: alpha, orange: beta, grey: gamma; bin width differs for each frequency band). (right) Linear regression of peak frequency per area and the radius r from the centre of the brain. The peak frequency decreases the closer an area is to the centre of the brain. Areas in the middle of the brain mostly show peaks in the delta band, whereas higher peak frequencies were found in areas further away from the centre. (TIF) [file pbio.1002498.s015.tif]
