# Supplementary material for: Individual Human Brain Areas Can Be Identified from Their Characteristic Spectral Activation Fingerprints
Source: PLoS Biol. 2016 Jun 29;14(6):e1002498. doi: 10.1371/journal.pbio.1002498 (PMC4927181; doi:10.1371/journal.pbio.1002498)
Supplement: S1 Text — Analysis of peak frequencies with strongest power for all brain areas. (DOCX) [file pbio.1002498.s016.docx]

**Supporting Information**

**Peak Frequency per Anatomical Area**

The spectral mode with the strongest power was most often the alpha band, usually found in posterior areas, followed by the delta band, often found in anterior and inferior areas (see S12 Fig). We also found beta-band modes, predominantly in central areas. The distribution of peak frequencies in terms of power was best described as a function of the radius *r* from the centre of the brain; the logarithmic peak frequency increased linearly with increasing distance from the centre of the brain, *R*^2^ = .16, *F*(1,113) = 20.97, *p*_corrected_ < .001. In other words, areas near the centre of the brain show more activity in lower bands, whereas areas on the cortical surface tend to show activity in higher frequency bands. The topography of peak frequencies is mostly consistent with previous findings of power distributions in the alpha, beta, and delta bands [1-3].

**References Supporting Information**

1. Hillebrand A, Barnes GR, Bosboom JL, Berendse HW, Stam CJ (2012) Frequency-dependent functional connectivity within resting-state networks: an atlas-based MEG beamformer solution. Neuroimage 59: 3909-3921.

2. Congedo M, John RE, De Ridder D, Prichep L (2010) Group independent component analysis of resting state EEG in large normative samples. Int J Psychophysiol 78: 89-99.

3. Chen AC, Feng W, Zhao H, Yin Y, Wang P (2008) EEG default mode network in the human brain: spectral regional field powers. Neuroimage 41: 561-574.
